# Model ensembles of ecosystem services fill global certainty and capacity gaps

**DOI:** 10.1126/sciadv.adf5492

**Published:** 2023-04-07

**Authors:** Simon Willcock, Danny A. P. Hooftman, Rachel A. Neugarten, Rebecca Chaplin-Kramer, José I. Barredo, Thomas Hickler, Georg Kindermann, Amy R. Lewis, Mats Lindeskog, Javier Martínez-López, James M. Bullock

**Affiliations:** ^1^ Net Zero and Resilient Farming, Rothamsted Research, Harpenden, Hertfordshire AL5 2JQ, UK.; ^2^ School of Natural Sciences, Bangor University, Bangor, Gwenydd LL57 2DG, UK.; ^3^ Lactuca: Environmental Data Analyses and Modelling, Diemen, Netherlands.; ^4^ UK Centre for Ecology and Hydrology, Wallingford OX10 8BB, UK.; ^5^ Department of Natural Resources and Environment, Cornell University, 226 Mann Drive, Ithaca, NY 14853, USA.; ^6^ Conservation International, 2100 Crystal Drive #600, Arlington, VA 22202, USA.; ^7^ Cornell Lab of Ornithology, Cornell University, 159 Sapsucker Woods Rd, Ithaca, NY 14850, USA.; ^8^ Global Science, Word Wildlife Fund, 131 Steuart Street, San Francisco, CA 94105, USA.; ^9^ Institute on the Environment, University of Minnesota, 1954 Buford Ave, St. Paul, MN, 55108, USA.; ^10^ Natural Capital Project, Stanford University, 327 Campus Drive, Stanford, CA, 94305, USA.; ^11^ Joint Research Centre, European Commission, Ispra, Italy.; ^12^ Senckenberg Biodiversity and Climate Research Centre, Frankfurt, Germany.; ^13^ Institute of Physical Geography, Goethe-University, Altenhöferallee 1, 60438 Frankfurt am Main, Germany.; ^14^ International Institute for Applied Systems Analysis, Laxenburg, Austria.; ^15^ Department of Physical Geography and Ecosystem Science, Lund University, Lund, Sweden.; ^16^ Department of Ecology, University of Granada, Avda. del Mediterráneo s/n, E-18006 Granada, Spain.; ^17^ Instituto Interuniversitario de Investigación del Sistema Tierra en Andalucía (IISTA), Universidad de Granada, Avda. del Mediterráneo s/n, E-18006 Granada, Spain.

## Abstract

Sustaining ecosystem services (ES) critical to human well-being is hindered by many practitioners lacking access to ES models (“the capacity gap”) or knowledge of the accuracy of available models (“the certainty gap”), especially in the world’s poorer regions. We developed ensembles of multiple models at an unprecedented global scale for five ES of high policy relevance. Ensembles were 2 to 14% more accurate than individual models. Ensemble accuracy was not correlated with proxies for research capacity, indicating that accuracy is distributed equitably across the globe and that countries less able to research ES suffer no accuracy penalty. By making these ES ensembles and associated accuracy estimates freely available, we provide globally consistent ES information that can support policy and decision-making in regions with low data availability or low capacity for implementing complex ES models. Thus, we hope to reduce the capacity and certainty gaps impeding local- to global-scale movement toward ES sustainability.

## Teaser

Global ensembles of ecosystem service models have increased accuracy and fill data gaps for less wealthy regions.

## INTRODUCTION

There is a burgeoning number of ecosystem service (ES) maps delineating an ever-growing understanding of the ways in which nature benefits people [e.g., (*1*, *2*)]. However, when ES data are available, they are typically inconsistent between countries, making standardized measurement or reporting difficult (*3*). Global maps (based on satellite and other data integrated in a variety of models) can provide readily available information when more locally relevant data are lacking (*4*), although it is questioned whether global maps provide accurate or useful information given their lack of sensitivity to local context (*5*). It is difficult to answer this question for most large-scale ES modeling exercises due to the lack of information on model accuracy, the closeness of the agreement between the modeled value and a reference value (*6*), the latter being considered “true” (*7*) even though the validation data are also often uncertain (*8*). Individual model performance varies, validation with empirical data is sometimes lacking, and results are typically reported without estimates of accuracy (*8*). Two key advantages of global maps are that they can fill gaps in data-poor contexts until local data can be collected or created, and they are consistent among countries (*4*). For example, at a local level, the Critical Ecosystem Partnership Fund made conservation investment decisions in Madagascar based, in part, on local information on the relative importance of sites for ES derived from models and globally available data (*9*). At a global scale, consistent data can be used for international policy and decision-making [e.g., informing targets and investments in the United Nations (UN) Sustainable Development Goals, the Convention on Biological Diversity post-2020 Biodiversity Framework, the UN’s System of Environmental-Economic Accounting-Ecosystem Accounting (*10*)]. Global data can also provide consistent and comparable local reporting for these international agreements, as well as broader context for local decisions by revealing wider regional, continental, and global patterns in ES status and trends (*4*).

Several studies have validated models of single ES [e.g., (*11*, *12*)] and rarely multiple ES [e.g., (*8*, *13*)]. Independent evaluations of models have often been unable to demonstrate the consistently superior accuracy of any individual model (*8*, *13*). While a few studies find that, on average, more complex ES models show better fit to validation data, the best-fit model varies regionally and often according to the validation data used (*8*, *13*). Thus, decisions based on a single model for an ES are less likely to be robust and, when models are in disagreement, it is difficult for practitioners (those engaging with information from ES models) to know which model should be used to support decisions (*14*). Projections by alternative models can be so variable as to compromise even the simplest assessment and therefore challenge the common practice of relying on a single method (*15*). This “certainty gap” greatly reduces the confidence that practitioners have in projections from ES models (*16*).

The certainty gap is unlikely to be uniformly distributed across the globe. In developing countries, reliable information about ES is critically important because the rural and urban poor are often the most dependent on ES (directly or indirectly), both for their livelihoods and as a coping strategy for buffering shocks (*17*). ES declines driven by overexploitation, habitat conversion, or climate change therefore undermine 80% (35 of 44) of the Sustainable Development Goals (*18*). However, ES data and accuracy estimates are often unavailable in developing nations, or in less affluent regions within nations, where they are most needed (*17*). There is an urgent need for evaluations of model accuracy to better inform decision-making, a need that has been emphasized by the Intergovernmental Science-Policy Platform on Biodiversity and Ecosystem Services (IPBES) (*19*). To address this, researchers have established standards for best practice using model-data (*8*) and model-model (*13*, *20*) comparisons to provide robust and transparent evaluations of accuracy. For example, an ensemble of models is more accurate, on average, than one model for any location, although the amount of improvement depends on the local context and the models used (*13*, *15*, *20*). However, while model ensembles are common in climate modeling and other disciplines (*15*, *21*), they have been largely neglected in ES studies (*22*). Simple (“committee average”) ensembles have been found to be at least 5% more accurate than individual ES models (*13*), while more complex, weighted ensembles provide even better predictions (up to 27% more accurate) (*20*). Furthermore, variation among models can provide an indicator of the uncertainty of the modeled ES estimate when no other information is available (*13*).

While using ensembles of ES models is possible, there are barriers that need to be overcome before it can become standard practice within ES science. Implementing multiple ES models remains a difficult undertaking for many researchers and practitioners (*13*). Barriers include lack of input data, resources, and capacity for data collection or collation and for modeling (*13*, *14*). As with the certainty gap, these barriers are typically more substantial in poorer nations. For example, creating ensembles of carbon storage models across three major platforms, ARIES (*23*), InVEST (*24*), and Co$ting Nature (*25*), requires access to the internet, high-quality input data, computational power, and geographic information systems (GIS) proficiency, as well as funds to support model subscription fees (where required) and the person-time required to learn and run three different models (*13*). Such resources can be out of reach for many researchers and practitioners. Furthermore, if practitioners must choose between running multiple models for a single service versus modeling additional services, then the former may be of low priority; thus, the widespread use of ES ensembles may be an unrealistic goal (*13*, *14*, *20*). We refer to the lack of these resources as the “capacity gap.” One potential solution to the capacity gap is that those who have the resources to create ES ensembles make the resulting data, as well as estimates of uncertainty, freely available [e.g., (*13*, *20*)].

**Fig. 1.**
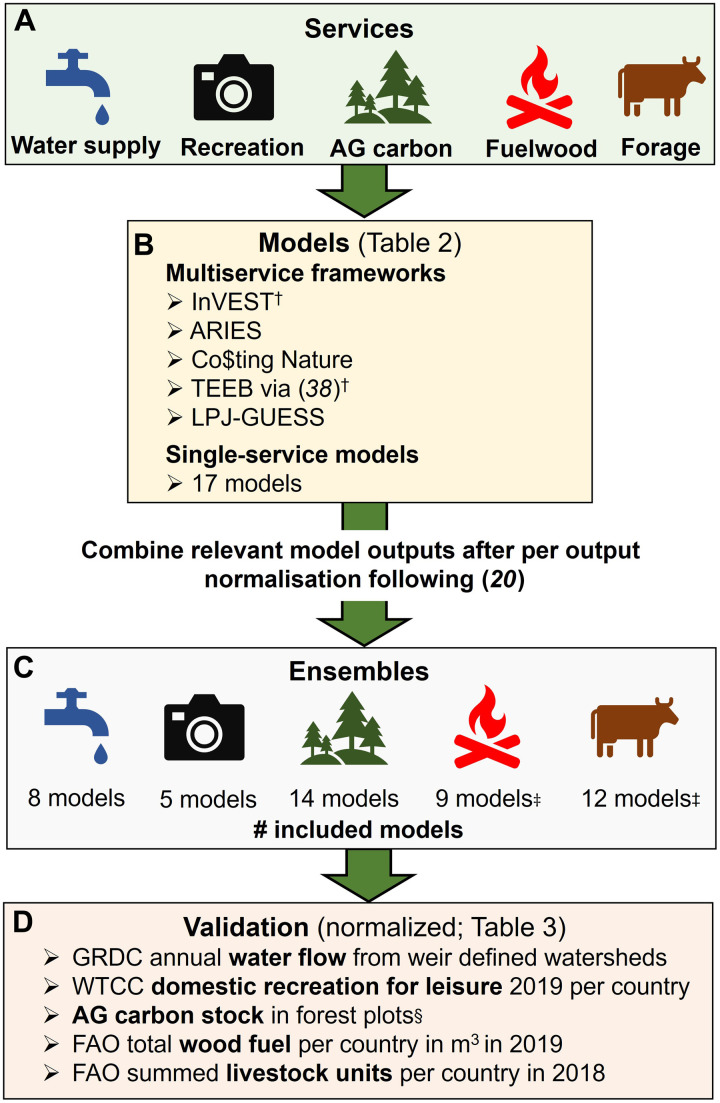
Schematic overview of the model flow, ensemble creation, and validation processes implemented in this study. We modeled five ES: potential water supply as flow in rivers, recreation as the number of visitors, potential aboveground (AG) carbon stock, potential fuelwood, and potential forage production capacity (**A**). We used models for these ES from five multiservice frameworks (i.e., multiple ES per modeling framework) and 17 individual ES models (Table 2) (**B**). These models are combined into model ensembles following Hooftman *et al.* (*20*), with the number of models in the ensemble for each ES shown in (**C**). We use validation data on each ES to test accuracy of the ensembles to both their own service and as proxy for other services (**D**). Symbol key: †Including choice of input data; ‡including models created by masking of AG carbon models with woody (fuelwood) or grassland land use masks [see (*8*) and section S3]; §combined pan-tropical biomass reference data (*38*) and U.K. (temporal) AG biomass stocks in forest estates (*20*). See Materials and Methods for full details.

To address the certainty and capacity gaps, we developed ensembles of models for five ES (Fig. 1) of high global and local policy relevance (*14*) and for which there are both: (i) a variety of models available that are feasible to run at a global scale and (ii) accessible, independent validation data to assess ensemble accuracy. We included three material services (water supply, eight available models; fuelwood production, nine models; and forage production, 12 models), one regulating service [aboveground (AG) carbon storage, 14 models], and one nonmaterial service (recreation, five models). Some of these ES are potential services (e.g., water, fuelwood, and forage) and some are realized (e.g., carbon recreation), where potential ES are “the outcomes from ecosystems that directly lead to good(s) that can be used and valued by people (e.g., harvestable products, and water supply), noting that some ESs can be both ecosystem processes and potential ESs” and realized ES are “all use and nonuse, material and nonmaterial outputs from ecosystems that are used and valued by people” (*26*, *27*). Both potential and realized service metrics are useful to support decision-making, with the latter providing insight into how the well-being of people is improved by nature and the former indicating that the maximum capacity of these potential well-being increases (*14*). We used model output predictions and created ES ensembles at an unprecedented global extent and at a 0.008333° resolution (approximately 1 km at the equator). We address the capacity gap by making the ensemble model outputs freely available (https://doi.org/10.5285/bd940dad-9bf4-40d9-891b-161f3dfe8e86) and providing the code (github.com/GlobalEnsembles and https://doi.org/10.5281/zenodo.7687580) to make the overall approach more accessible. To address the certainty gap, we tested the accuracy of these ensembles against independent validation data (including country-level statistics and actual biophysical measurement), and investigated spatial patterns in ensemble accuracy.

## RESULTS

**Fig. 2.**
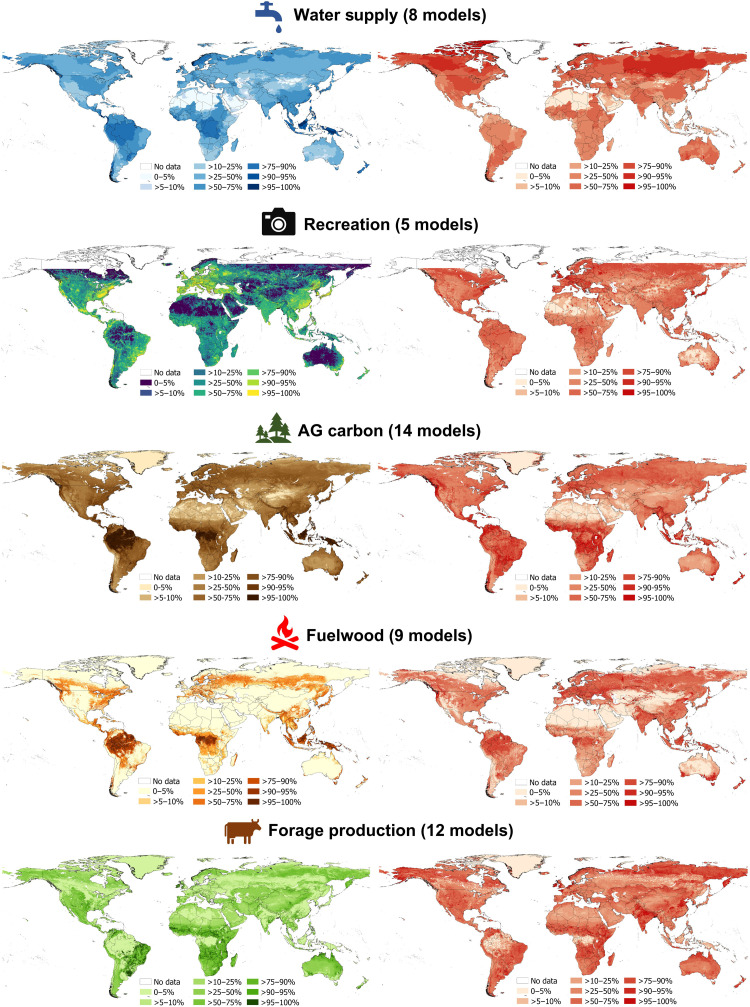
Median ensemble values and associated standard error of the mean. Left: Median ensembles values from models for five ES of high policy relevance (*14*). We created ensembles for water, recreation, AG carbon, fuelwood, and forage production at global scale and at an 0.008333° resolution by taking the median value of multiple models for each grid cell. Addressing the capacity gap, we make these freely available via https://doi.org/10.5285/bd940dad-9bf4-40d9-891b-161f3dfe8e86 and maps produced using alternative ensemble approaches (including mean, PCA, correlation coefficient, and regression to the median and leave-one-out cross-validation log-likelihood approaches; see the Supplementary Materials). Right: Addressing the certainty gap, we show the SEM associated with each ES ensemble output, which, in accordance with previous research (*13*), our investigations show can be used a proxy for ensemble accuracy in absence of validation data (fig. S4). All maps scaled in deciles 0 to 100%. True zero values (colored) are distinguished from no data (white). Selected case study regions are shown in section S6. The figures are available via https://github.com/GlobalEnsembles/Maps and https://doi.org/10.5281/zenodo.7687580, and the data are available via https://doi.org/10.5285/bd940dad-9bf4-40d9-891b-161f3dfe8e86.

**Fig. 3.**
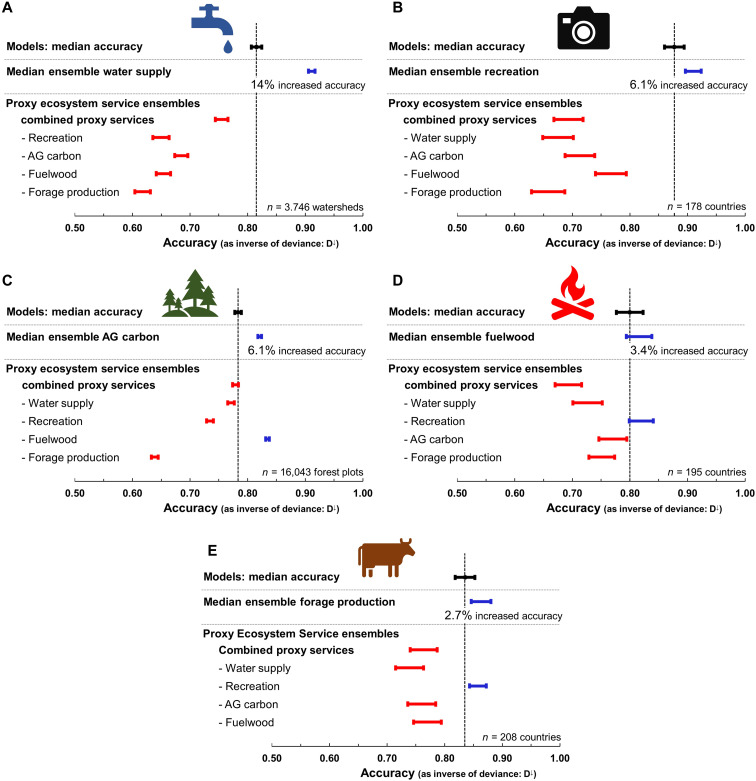
ES ensembles show increased accuracy when compared to individual models. Shown are the median ensembles for: (**A**) water, (**B**) recreation, (**C**) AG carbon, (**D**) fuelwood, and (**E**) forage production. ES theory on bundles suggest that values for different ES can be spatially related to each other, either positively or negatively (*2*). However, spatial correlations among ES, while they do occur, may vary geographically, meaning that there is no consistent correlative relationship among ES over large spatial scales (*29*). To test this, we spatially correlated each global ES ensemble output with the output of all other ES ensembles, both as a group (or bundle; i.e., for all ES ensembles combined) and for each ES individually. Our results showed ES bundles to be a relatively poor predictor of an additional ES and that most ES ensembles were not well correlated with other ES on an individual ES basis. Vertical dashed lines indicate the among-run median accuracy of an individual model chosen with no a priori information (i.e., at random). Blue bars indicate that a model (or ensemble) accuracy was significantly higher than the median accuracy of the models (length of bars represent among-model SD). Red bars indicate that accuracy was significantly lower than the median of the models.

Here, we present results using an unweighted median ensemble (*8*) approach (i.e., taking the median value of multiple models for each grid cell; Fig. 2). Other ensemble approaches, including unweighted (mean), and weighted [deterministic consensus, principal components analysis (PCA) and correlation coefficient; iterated consensus, regression to the median and leave-one-out cross-validation log likelihood) approaches (*20*), which give consistent conclusions, are described in the Supplementary Materials. When compared to independent validation data (Fig. 1), global ES ensembles were more accurate than an individual model chosen at random (Fig. 3 and Table 1). Median ensemble improvement per validation datapoint for each ES was 14% for water (resolution of the validation data, weir defined watersheds), 6% for recreation (national scale), 6% for AG carbon (plot scale), 3% for fuelwood (national scale), and 3% for forage production (national scale; Fig. 3 and Table 1). Thus, using global ES ensembles rather than an individual ES model reduces the certainty gap for practitioners with no a priori information on model accuracy. In general, the weighted ensembles provided more accurate predictions than unweighted ensembles (figs. S15 and S16) and so should be favored by practitioners. Ensembles further address the certainty gap by transparently conveying any spatial variation in accuracy. For example, the SE of the mean associated with each ES ensemble (Fig. 2) correlates with the accuracy of the ensemble and so can be used as a proxy for ensemble accuracy in absence of validation data [(*13*) and fig. S4], indicating the accuracy of the ensembles in any specific geographic location. Our results are consistent when using alternative accuracy metrics (e.g., Spearman’s ρ; see section S5).

**Table 1.** One-tailed correlations as *F* values with significance of the inverse of deviance per validation datapoint (where increasing accuracy is represented by increasing the inverse of deviance) of the five ES ensembles against globally available metrics that could potentially impact model accuracy. One-tailed tests were applied to test the hypothesis that the ensemble accuracy increases with higher values of each development/equality measure (two-tailed is presented in table S7, including effect sizes). Degrees of freedom were standardized at 178 following a bootstrap convergence model for all services. Significance of the presented *F* values were assessed taking account of multiple tests, using Hochberg’s step-up correction with eight tests per ES. An interaction model is added testing for interactions between GDP per capita and income equality, reflecting that income may be better represented using both mean and variance. To conform to the normality assumptions of the analysis, all metrics were arcsine transformed, with the exception of GDP per capita, which was log_10_ transformed, and the Human Development Index (HDI), which was not transformed. See Table 3 for the sources of each validation dataset.

	Water supply	Recreation	AG carbon	Fuelwood production	Forage production
**Accuracy improvement (inverse of deviance)** Ensemble versus a random selected model (median among models)†	14%	6.1%	6.1%	3.4%	2.7%
**Spatial Autocorrelation‡**	15.3***	14.6***	211***	0.47	0.14
**Development/equality per country**					
GDP per capita	1.38	<0.01	1.21	3.58	0.24
Human Development Index	1.51	<0.01	0.14	6.43	0.25
Income equality (Gini index)	0.17	6.69	1.37	<0.01	0.71
% People in R&D	1.44	<0.01	0.15	4.85	0.08
% GDP to R&D	0.08	<0.01	0.14	3.79	0.37
**Interaction model**					
GDP per capita	1.76	0.18	0.16	1.29	0.02
Income equality	1.67	0.22	0.16	0.50	0.02
GDP × income equality	0.06	0.34	1.04	0.16	2.67

†Mean of pairwise comparisons per 1000 bootstrap runs.

‡Two-sided tested without direction.

***P < 0.001 corrected.

While the results presented here show that ES ensembles reduce the certainty gap, differences in ensemble performance between regions or countries might be expected. For example, nations investing more in research capacity might have better input data or have more researchers who develop and test ES models, potentially resulting in model outputs that are more locally relevant in those areas (*28*). Thus, we might expect that ES ensembles perform better in countries with higher GDP, Human Development Index (HDI) scores, or research capacity. After accounting for spatial autocorrelation (see Materials and Methods) and applying the Hochberg correction to account for multiple tests, we found no evidence that ensembles are more accurate in countries with higher GDP (even when accounting for within-country variability using Gini metrics of inequality), with higher HDI, or with higher research capability (expressed as the percentage of people who are researchers and proportion of GDP invested in research; Table 1). The results are consistent when using alternative statistical approaches (tables S7 to S9). These findings suggest global consistency in ensemble accuracy, in relation to the potential drivers of variation that we tested (Table 1). A potential caveat is that if the validation data themselves are biased (for example, less accurate across developing countries), then true patterns in ensemble model accuracy could exist undetected.

Last, while the five ES ensembles made available here contribute to addressing the capacity gap, practitioners will often require accurate ES information on many additional services, including many for which there are no models (*14*). ES theory on bundles suggests that values for different ES can be spatially related to each other, either positively or negatively (*2*). However, spatial correlations among ES, while they do occur, may vary geographically, meaning that there is no consistent correlative relationship among ES over large spatial scales (*29*). To test this, we spatially correlated each global ES ensemble output with the output of all other ES ensembles, both as a group (or “bundle;” i.e., for all ES ensembles combined) and for each ES individually. Our results showed ES bundles to be a relatively poor predictor of an additional ES (Fig. 3). Similarly, most ES ensembles were not well correlated with other ES on an individual ES basis.

## DISCUSSION

To help fill a major capacity gap in terms of available ES information for many countries, we have provided globally consistent ensemble data on five ES (https://doi.org/10.5285/bd940dad-9bf4-40d9-891b-161f3dfe8e86) and the code required to produce them (github.com/GlobalEnsembles and https://doi.org/10.5281/zenodo.7687580). Finding increased performance through use of ensemble approaches is common in other fields (*20*), although an increase is not universal (*30*). Because of underlying assumptions, model predictions (including those from ES models) are all potentially biased in direction and amount, with biases varying among models due to their specific construction and available input data (*20*). The improvement in accuracy when using ensembles likely derives from suppression of idiosyncratic differences by inclusion of multiple possible system representations (termed as “portfolio effect”), providing a more reliable average estimate (*20*, *31*). However, this effect is lessened if assumptions, and therefore concomitant biases, are shared across models (*20*). This highlights the importance of including: (i) multiple model outputs in model ensembles (*32*), including from models not explicitly identified as ES models, such as hydrological models (*20*); and (ii) where data are available, model validation (*8*); see Dormann *et al.* (*31*) and Hooftman *et al.* (*20*) for further theoretical explorations. Using ensembles also improves consistency across independent studies. For example, considering two studies applying different models in different locations, it is uncertain how comparable the findings are (*4*). However, if both studies use model ensembles, even if the ensemble approaches are not identical, then results will be more comparable. This is because variation among ensemble approaches is substantially lower than among individual models (*20*), resulting in greater consistency and coherence. Thus, potential applications of ES ensembles include supporting nations’ efforts to implement natural capital accounting (*3*).

Our finding that global ES ensembles perform just as well in less wealthy regions with lower research capacity, where this information is often most needed, emphasizes the utility of these modeled data. This might reflect that ES models are increasingly tested and parameterized using global-scale Earth Observation data. In addition to the ensemble maps themselves, we provide estimates of accuracy (https://doi.org/10.5285/bd940dad-9bf4-40d9-891b-161f3dfe8e86). The ability to quantify accuracy when it comes to ES is often lacking and, at worst, this can result in perverse outcomes, with the “pot luck” associated with model selection (i.e., without a priori accuracy information) sometimes resulting in implementation of low-accuracy outputs and suboptimal decisions (*8*, *19*). For policy and decision-making, accuracy estimates are as important as the ES maps themselves, and the lack of information about uncertainty is one driver of the “implementation gap” between ES research and its incorporation into policy and decision-making (*16*). By providing accuracy maps, we are directly addressing this certainty gap. However, future work should seek to improve on these accuracy maps, in particular through the collection and inclusion of additional validation data at local scales, as using the national- and watershed-scale validation data that are currently available may be a poor proxy of model accuracy at local scales.

Important capacity gaps remain. Most ES research predominantly focuses on a limited set of material and regulating services because the data are widely available, and their underlying processes are relatively well understood (*33*). This means that our current ability to assess or predict unmodeled ES is low. We found that ensembles, whether as an individual ES or as a bundle, do not accurately predict other ES at global scales. It could be that as more ES are included in a bundle, predictive power of the bundle for unmodeled ES improves; in a recent analysis, global maps resulting from individual models for 12 ES show high correlations between any one service and the remaining 11 (*2*). This is possibly because the more and more diverse ES that are included, the more likely that unmodeled ES will also be represented by the same set of ecosystems, either because they are similar to modeled ES or simply by chance. In general, the utility of the bundle approach is debated, with Spake *et al.* (*29*), suggesting that a hypothesis-driven approach is required to predict relationships between ES. Ultimately, while individual models are available for more ES than are presented here, model development is urgently required before ensembles of additional ES can be assessed.

Practitioners show both capacity and willingness to engage with accuracy information when it is made available (*14*). Accuracy estimates allow practitioners to determine what level of confidence is acceptable to them and to use their own expertise to make potentially contentious decisions (*34*). Given limited resources, accuracy information can play an important role in prioritization. For example, the accuracy of estimates may be vital in distinguishing between two sites with high levels of ES production. Another example could be a decision to give a site with high accuracy of medium ES levels lower priority over a potentially high-value site with medium or low accuracy; this is contentious but defensible if accuracy information is transparently conveyed to practitioners. Thus, providing estimates of accuracy should become standard practice within the ES community (*22*). High levels of inaccuracy or uncertainty of ES estimates should not lead to inaction but instead highlights the risks of making decisions using poor data, what data may need to be gathered to improve model inputs, or the need to develop new or improve existing ES models. The model-estimated quantity of ES and its accuracy should not be the only metrics considered in decision-making. For example, as the well-being of some marginalized groups may depend on ES where models or data are lacking, or uncertainty is high, therefore it is critical to incorporate local knowledge and values in any decision-making process (*2*). Model accuracy is one of a range of metrics considered by practitioners when determining whether model outputs can be used to support decision-making, with others including spatial resolution and the ability to incorporate scenarios (*14*). Thus, simply reducing uncertainty is not necessarily going to lead to better policy decisions. However, in regions with a large capacity gap, practitioners lack any comprehensive spatial data on most ESs. For these regions, our 1-km^2^-resolution ES ensemble outputs provide, at a minimum, some data with a level of validation and associated accuracy at little to no cost to the practitioner (*14*).

We conclude that ensemble modeling of ES can help reduce capacity and certainty gaps by, for example, making more accurate ES estimates freely available. We suggest ES scientists adopt ensemble approaches (shown here to be, on average, a more accurate approach than using individual models) and accompany model outputs with estimates of uncertainty. These changes may help reduce the implementation gap between ES research and policy and decision-making (*14*, *33*), in particular for assessments by IPBES and the Intergovernmental Panel on Climate Change.

## MATERIALS AND METHODS

**Fig. 4.**
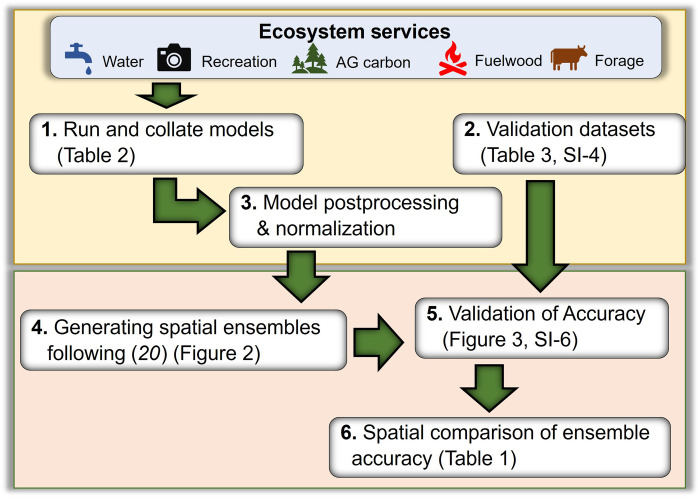
Schematic representation of our analysis, with arrows showing information flows. Numbers represent the steps within our methods; input tables and result figures are indicated.

We developed and tested (against validation data) ensembles of models for five ESs (Figs. 1 and 4) for which there are both a variety of models which are feasible to run at a global-scale (*8*, *20*) and accessible independent validation data. We used model output estimates of ES (listed in Table 2) to create ensembles and then validated them against independent data (Table 3) using methods developed previously for United Kingdom (*20*) and sub-Saharan Africa (*8*). To ensure comparability among model outputs, we standardized them by normalizing outputs from individual models before creating ensembles, following the same procedure for the validation data. We explored the spatial variation in accuracy of ES ensembles, using a variety of metrics. Last, we investigated the use of ES ensemble “bundles” as proxies for other ES. We depict our overall process in Fig. 4 in six steps. Our calculations were performed using Matlab v7.14.0.739, ArcMap 10.7, and ArcPro 2.7, using Arcpy coding for loops. Relevant code can be found at github.com/GlobalEnsembles and https://doi.org/10.5281/zenodo.7687580.

**Table 2.** Summary information for the individual ES models used in this study.

Model	ES	Details	Model output resolution
**Multiservice frameworks**			
ARIES k.explorer (*23*) for year = 2020 (https://integratedmodelling.org/modeler)	Recreation†	Recreation run online per country; carbon follows (*52*); all in metric tons per hectare, except recreation in normalized no. of people (section S1-4)	0.008333°, mostly worldwide
	AG carbon		
	Forage production‡		
	Fuelwood production		
Co$ting Nature (*25*) (https://www.policysupport.org/costingnature)	Water supply	Run online as 10° tiles; subsequent among tile normalization; all unitless normalized indexes, except water in m^3^ per year.	0.008333°, not above 60° north
	AG carbon		
	Recreation†¶		
	Forage production		
	Fuelwood production		
InVEST v3.8.7 (*24*) (https://naturalcapitalproject.stanford.edu/software/invest)	Water supply	Desktop tool, parameterized for this project (section S1-1). Water supply in m^3^ per grid cell; recreation in number of photo uploads; carbon/forage/fuelwood in metric tons per hectare.	0.008333°, worldwide
	AG carbon		
	Recreation¶		
	Forage production‡		
	Fuelwood production§		
Lund-Potsdam-Jena GeneralEcosystem Simulator (LPJ-GUESS) (*53*)	Water Supply	Data set from (*8*) and run as described therein. Water supply in m^3^ per grid cell; carbon/forage/fuelwood in metric tons per grid cell.	0.5°, worldwide
	AG carbon		
	Forage production#		
	Fuelwood production§		
TEEB via Costanza *et al.* (*45*)	Water supply	In local GIS environment (section S1-2), all in US$ for the year 2007 as provided by (*45*)	0.002778°, worldwide
	AG carbon		
	Recreation		
	Forage production‡		
	Fuelwood production		
Scholes (*54*) via Willcock *et al.* (*8*), livestock distributions extended worldwide	Water supply	In local GIS environment extended from (*8*); section S1-3. Water supply in positive growth days; forage in livestock units per hectare.	0.008333°, worldwide
	Forage production		
**Single service models**			
Aqueduct Global Maps 2.1 (WRI) (*55*): accumulated water run-off (https://wri.org/data/aqueduct-global-maps-21-data)	Water supply	Existing data; as available blue water (m^3^) per catchment outlet	Watershed polygons,worldwide
European map of AG biomass stocks (*36*)	AG carbon	Existing data, from (*20*); as metric tons per hectare	0.008333°, Europe only
ESA CCI Biomass Climate Change Initiative (*39*) (data.ceda.ac.uk/neodc/esacci/-biomass/data/agb/maps/v2.0/geotiff/2018)	AG carbon	Existing data; as metric tons per hectare	0.0008888°, worldwide,forest only
FAO combined gridded livestock distributions (https://fao.org/livestock-systems/globaldistributions)	Forage production	Existing data, summed LSUs among types (section S2) per grid cell	0.08333°, worldwide
Integrated GEOCARBON global forest biomass (*38*) (https://www.wur.nl/en/Research-Results/Chair-groups/Environmental-Sciences/Laboratory-of-Geo-information-Science-and-Remote-Sensing/Research/Integrated-land-monitoring/Forest_Biomass.htm)	AG carbon	Existing data; as metric tons per hectare	0.01°, worldwide, forest only
Gilbert *et al.* (*43*); Combined gridded livestock distributions (https://dataverse.harvard.edu/dataverse/glw_3)	Forage production	Existing data, summed LSUs among types (section S2) per grid cell	0.08333°, worldwide
Global Forest Watch, AG biomass (*40*) (https://data.globalforestwatch.org/-datasets/above-ground-live-woody-biomass-density/data)	AG carbon	Existing data; as metric tons per hectare	0.00025°, worldwide, forest only
JRC Above ground Biomass (*37*) (https://data.jrc.ec.europa.eu/dataset/biomass)	AG carbon	Existing data; as metric tons per hectare	0.0008333°, Europe only
Chaplin-Kramer *et al.* (*2*)	Recreation	Existing data in number of people per grid cell	0.01667°, not above 60° north
WaterWorld (*35*): Accumulated water run-off (https://www.policysupport.org/waterworld)	Water supply	Run online per available catchment in m^3^ per catchment outlet	0.008333°, partially worldwide
WaterWorld (*35*): Water Budget per cell (https://policysupport.org/waterworld)	Water supply	Run online per available catchment in m^3^ per grid cell	0.008333°, partially worldwide
**Single-service carbon models with masked use for grazing and fuelwood**			
Avitabile *et al.* (*38*): Carbon in vegetation (https://www.wur.nl/en/Research-Results/Chair-groups/Environmental-Sciences/Laboratory-of-Geo-information-Science-and-Remote-Sensing/Research/Integrated-land-monitoring/Forest_Biomass.htm)	AG carbon	Existing data; as metric tons per hectare	0.008333°, tropics only
	Forage production‡		
	Fuelwood production§		
Conservation International Total Carbon in vegetation (*56*) (https://conservation.org/projects/-irrecoverable-carbon)	AG carbon	Existing data; as metric tons per hectare	0.002695°, worldwide
	Forage production‡		
	Fuelwood production§		
Kindermann *et al.* (*57*) AG biomass stocks	AG carbon	Existing data, from (*20*); as metric tons per hectare	0.008333°, worldwide
	Forage production‡		
	Fuelwood production§		
ORNL DAAC (NASA), AG biomass density (*58*) (https://daac.ornl.gov/cgi-bin/dsviewer.pl?ds_id=1763)	AG carbon	Existing data; as metric tons per hectare	0.002778°, worldwide
	Forage production‡		
	Fuelwood production§		

†Including postprocessing with a further data set (section S1-4); based on AG carbon with a ‡grassland and §woodland MODIS land cover mask following (*9*) (section S3).

¶Realized service based on photo uploads; # combined C_3_ and C_4_ carbon.

**Table 3.** The empirical validation datasets used in this study (mapped in section S4). ES models need to be evaluated against the real world to determine whether they are able to provide sufficiently accurate information for regional- or local-scale policy- and decision-making. Since the true value can never be absolutely determined, acceptable reference values must be used. Empirical data can be used as reference values to evaluate ES model accuracy (*8*). Although such reference values are likely to have errors associated with them and may not be totally representative of the true values (*8*), this approach is widely accepted in environmental sciences (*48*).

**Service**	**Validation set**	**Description**	**Original resolution**	**Details**
**Water supply**	Global Runoff Data Centre: River discharges	3746 selected stations. Mean annual water flow per hectare catchment (m^2^ ha^−1^)	Catchments as polygons (section S4)	Selected on still running after 2000 and containing at least 25 years of data (https://www.bafg.de/GRDC/EN/Home/homepage_node.html)
**Recreation**	WTCC: Tourism economic impact reports per country	Total GDP of domestic recreation for leisure in US$ for 178 countries	Country (GAUL-2) polygons	Country sheets for 2019, calculated as recreation GDP contribution (US$) × [% domestic spending × % leisure spending]. https://wttc.org/Research/Economic-Impact.
**AG carbon**	1. Pan-tropical biomass in forest plots	ABG stock in metric tons per hectare for 14,478 forest plots (*34*)	Point data	Tropical region. Pan-tropical biomass reference data https://www.wur.nl/en/Research-Results/Chair-groups/Environmental-Sciences/Laboratory-of-Geo-information-Science-and-Remote-Sensing/Research/Integrated-land-monitoring/Forest_Biomass.htm.
	2. U.K. carbon in forest estates	Mean ABG stocks in metric tons per hectare from 1606 estates	Forest polygons	Temperate region. Modified data taken from (*20*), original data from UK Forest Research
**Fuelwood production**	FAOStat: Wood fuel per country	Wood fuel in m^3^, summed nonconiferous and coniferous for 2019 for 195 countries	Country (GAUL-2) polygons	https://fao.org/faostat/en/#data/FO
**Forage production**	FAOStat: Livestock per country	Summed livestock units per country for 2018 for 208 countries	Country (GAUL-2) polygons	Animals are: Asses, buffaloes, camels, cattle, chickens, goats, horses, mules, pigs, and sheep. https://fao.org/faostat/en/#data/EK

### Run and collate models

We collated models for this study according to their availability and feasibility to be run at a global scale and to reflect different approaches to modeling ES, obtaining appropriate registrations and licenses if necessary. The collated models are summarized in Table 2, including their output grid sizes (spatial resolution), as well as whether the model outputs are existing [i.e., can be found online; e.g., (*10*, *30*)], are generated online (ARIES, Co$ting Nature, and WaterWorld) or can be calculated with a desktop tool (InVEST) or in local ArcGIS environment (Scholes and TEEB). For models that require input data choices (InVEST, Scholes, and TEEB), we refer to section S1 for details and supporting data. For models that were taken from Willcock *et al.* (*8*) and Hooftman *et al.* (*20*), we refer to the descriptions in those papers.

All model outputs were projected to WGS 1984 (EPSG 4326) and rescaled to a 0.008333° grid (approximately 1 km at the equator), resampling models where necessary (Table 2). In general, when upscaling, cells were aggregated by calculating the mean of the grid cell values with no-data cells ignored; when downscaling, ArcPro’s bilinear recalculation algorithm was used for resampling. This latter resampling resulted in a smooth transition by assuming values of smaller cells via linear extrapolations from neighboring cells (e.g., for LPJ, gridded livestock). Small-scale nonlinearity (e.g., as a result of unmodeled features) is not included in this downscaling; hence, an output would heavily depend on postprocessing assumptions and inputs and be a model in its own right. Rescaling factors are not needed during these calculations since these will not change relative values (i.e., resulting from subsequent normalization; step 3). All outputs were clipped and aligned to the exact same extent with standard number of rows and columns (43,200 × 18,600), using ArcPro’s bilinear recalculation algorithm.

While all model outputs were obtained at global scale, not all cover the entire globe (Table 2). Only the terrestrial globe was considered, but there were other specific constraints. For example, servers for certain online models restricted overly large data flows. Specifically, ARIES k.explorer was not able to run the recreation module per country for North America and parts of Europe because of the high level of detail in the supporting maps (*23*); WaterWorld was not able to run the largest watersheds such as the Amazon basin, the Mississippi, and the Yangtze (*35*). Furthermore, Co$ting Nature is limited to latitudes below 60° north due to lack of input data for northern regions (*25*). We used AG carbon models that were region specific, two for Europe (*36*, *37*), one for the tropics (*38*), and three that were forest vegetation specific (*38*–*40*).

### Validation datasets

Our validation datasets are listed in Table 3 (and mapped in section S4). Broadly, they include either informed expert statistics [such as country-level statistics from the FAO (forage production and fuelwood) and recreation values from the World Travel and Tourism Council], or actual biophysical measurement (tree inventory plots for AG carbon, and weir data for water flow):

• Our water supply validation dataset is catchment based. Specifically, we used a Global Runoff Data Centre (GRDC) dataset with 3746 weirs (Table 3 and fig. S1), covering all regions but not all land area. For each weir, bespoke catchments polygons were delineated using the a 90-m SRTM digital elevation map (*41*), following Willcock *et al.* (*8*).

• The recreation validation data consisted of the 178 available country sheets for economic and employment impact of travel and tourism of the World Travel and Tourism Council for 2019 (i.e., pre-COVID), providing the estimated total GDP of Tourism and Travel in U.S. dollars. It also contains estimates for the proportion spent on business and leisure and the proportion that is from domestic and international tourism. Three of the five recreation models represent leisure-oriented local access to nature, including gravity models (*42*). Therefore, to use validation data comparable to our modeled outputs, we multiplied the Tourism and Travel GDP with the proportions for leisure and domestic to get to “GDP of domestic recreation for leisure.”

• The AG carbon validation data are a combination of pan-tropical biomass in forest plots from ForestPlots.Net (*38*) and from the U.K. assessment of carbon in all forest estates (*20*). By using both datasets, we are able to validate the models in both temperate and tropical contexts. See Avitabile *et al.* (*38*) and Hooftman *et al.* (*20*) for further details.

• The fuelwood and forage production validation data are country-level statistics from FAO for 2019 (195 countries available) and 2018 (208 countries), respectively (fig. S2).

Each dataset has associated uncertainties (*8*), but after an extensive review of data, we identified these as the best-suited reference values for validation [i.e., metrics that corresponded most closely to those modeled, have been published in the peer-reviewed literature and/or widely accepted as an authoritative source (e.g., FAO statistics), and are available globally or for a large number of countries]. Both model and validation data are normalized (see below) to ensure comparability and remove unavoidable differences in exact units. The validation data are as independent as feasibly possible from the models; however, because of data deficiency, some aspects of an individual model may have been trained with local census data, which could, in part, relate to validation data. For example, gridded livestock from FAO and Gilbert *et al.* (*43*) are trained on various regional census data, some of which may have been included in the national-scale forage production validation data, and some of the plots from Avitabile *et al.* (*38*) may have been used to estimate the carbon stocks per land cover class per ecofloristic zone as used in ARIES (*23*).

### Model postprocessing and normalization

General model output postprocessing included projecting to WGS 1984, rescaling and clipping to the specified extent (step 1), and detecting and masking no-data values. The latter was especially applicable for forest only biomass/carbon data sets (Table 2) and for sea/large water bodies in various model outputs. In making ensembles, true zeros contribute to the average, whereas no-data are ignored. Postprocessing of ARIES and Co$ting Nature model outputs with additional data (marked † in Table 2) is discussed in section S1-4. This includes the procedure of among tile rescaling of Co$ting Nature, as the framework produces outputs in 10° tiles that are individually normalized. Therefore, tiles need to be rescaled using other global-scale estimates (section S1-4). AG carbon model output postprocessing with Moderate Resolution Imaging Spectroradiometer (MODIS) land cover (*44*) masks into forage production and fuelwood outputs following (*8*) as detailed in section S3.

To ensure comparability among model outputs, we standardized by normalizing each individual model output before making ensembles. This normalization followed (*13*, *20*) and allowed us to address differences in units among models, such as monetary benefit transfer versus satellite-based tree cover densities or water run-off, and negates the need for conversion factors (e.g., between biomass and AG carbon). To avoid impacts of extreme values without eliminating these datapoints, we used a double-sided Winsorising protocol for normalization (*20*), using the values associated with the 2.5 and 97.5% percentiles to define the minimum (0) and maximum (1) values (values below or above these percentiles became 0 or 1, respectively). Winsorising loses the extremes and so does curtail skew but avoids influences of very large and very small values (*20*). The Winsorising procedure can be found can be found at our GitHub account (github.com/GlobalEnsembles/Winsorising), both as Matlab and Arcpy coding. The validation datasets were subjected to the same Winsorising protocol. It must be noted that, even when modeling the same ES, many of the ES models estimate different constructs to some extent, often with varying units (Table 2). However, since our statistical analyses focused on relative ranking, it is unlikely that these uncertainties impacted our findings greatly [see (*8*) for a full discussion].

### Generating spatial ES ensembles

The procedures to generate different types of ES model ensembles are discussed in Hooftman *et al.* (*20*). Here, we focus on an unweighted ensemble, which is the median value of the model outputs calculated per grid cell. A selection of weighted methods developed by (*20*) (including mean, PCA, correlation coefficient, regression to the median, leave-one-out cross-validation, and log-likelihood approaches) is reported in section S7. These alternative ensemble approaches show consistent patterns and comparable accuracy to the relatively simple median ensemble.

For recreation, AG carbon, fuelwood, and forage production, our ensembles were based on per-grid cell estimates of the respective model outputs. Here, models for AG carbon, fuelwood, and forage production are comparable point-based estimates of local resources, although differing in complexity and initial assumptions (*8*, *20*). In addition, our recreation ensemble comprises different modeling methods that provide comparable predictions of potential recreational pressure: observations [photo uploads: (*24*, *25*)], population movement through gravity functions (*2*, *23*), and benefit transfer (*45*); see section S1-4 for a full discussion. For water supply, our ensembles are accumulated flow estimates following the global HydroSHEDS catchments definition (*46*). For grid cells, ensembles were created using ArcPro Cell Statistics module, with the median or SD as the input statistic. Because of the way certain models accumulated water flows (WaterWorld and Aquastat) a per-grid cell approach was not possible for water supply, so the sum of grid values within catchment polygons was calculated for each catchment. In the case of accumulated flow models (WaterWorld and Aquastat), we used the maximum value per polygon assumed to be the flow out of the HydroSHEDS pour point. Since HydroSHEDS information do not contain the spatial location of the exact pour point, we could not correct for differences in routing information as we do for the GRDC validation catchments (step 5). We used a forced 0.001° grid size to minimize edge effects.

As all models are normalized to the same 0 to 1 scale, calculations do not require any additional scaling factors. The spatial representation of the ensembles and variation are generated on the same extent and grid as described under step 1 and can be downloaded from the Environmental Informatics Data Centre (https://doi.org/10.5285/bd940dad-9bf4-40d9-891b-161f3dfe8e86). The water ensembles are there available as HydroSHEDS (*46*) defined accumulated water flow (vector format), the other four ES as geotiffs (raster format). Since not all model outputs are globally comprehensive, variation is expressed by a SEMs as $\left(\frac{\sigma_{\left(x\right)}}{{\sqrt{n}}_{\left(x\right)}}\right)$, instead of SD (σ), with *n* the number of models per grid cell *x*. The ensembles are renormalized to represent the full 0 to 1 range.

### Validation of accuracy

After creating the ensembles, the model and ensemble outputs were calculated at the spatial resolution of the validation data. For recreation, fuelwood, and forage production, the validation data are available on a per-country basis, so this was done by calculating the sum of all model ensemble grid cells within countries. Country definitions followed FAO Global Administrative Unit Layers (GAUL) level 2 with 2014 definition. This map includes separate polygons for overseas territories. When overseas territories were treated separately in one of the validation data sets [e.g., Martinique (FR) or British Virgin Island (UK)] those values were extracted as separate datapoints from the ensembles. We refer to all these spatial units as “countries,” although not all units have that designation. For each individual model, outputs were obtained for each country polygon with the ArcGIS Zonal tool with a forced 0.001° grid size to minimize edge effects, i.e., all predicted values were obtained by downsampling into 0.001° grid cells. For AG carbon plots, the point-based location of the forest plot was used as the mean value of underlying 0.001° grid cells. For grid-based water supply estimates, the sum of grid values per watershed polygon was used. In the case of accumulated flow models (WaterWorld and Aquastat), we corrected for potential small-scale differences in flow routing among these models by taking the maximum flow value within a 0.041665° range (five cell widths) of the GRDC-reported location of the weir station (*20*), without exceeding the aligned watersheds. We note that these validation data are diverse (Table 3), being collected using a range of methods of varying reliability, including expert opinion (e.g,. country-level statistics from the FAO) and biophysical measurement (e.g., tree inventory plots and weir data on water flow). Hence, each dataset has associated uncertainties (*47*) but, since the “true value” can never be absolutely determined, provides useful reference values for validation (*8*, *13*, *48*). However, given that the datasets covered a wide range of methods and our focus was on ranked correlative relationships (below), there is unlikely to be systematic bias and so data quality issues should have a low impact on our results. We refer to references (*8*) and (*13*) for a full discussion of ES model validation.

To create ES ensemble proxy services, we followed the procedure as above, e.g., AG carbon summed per country to compare to national-scale validation data, recreation, forage production, and fuelwood summed within catchments (for comparison to global runoff data), and at the point location of the forest plots (for comparison to AG carbon data). To be able to use accumulated water flow as proxy for country-validated services, we split the HydroSHEDS by countries, generating subcatchments where they crossed borders. Following this, data extraction and ensemble procedure was followed anew as described above. Similarly, for forest plot locations, water flow ensembles were generated for the plot locations only.

Ensemble, bundle, and model output accuracy was assessed following the inverse of the deviance (*D^↓^*) as was developed in (*8*) following

$$D^{↓}=1-\left(\frac{1}{n}\times \sum_{x}^{n}|X_{\left(x\right)}-Y_{\left(x\right)}|\right)$$ (1)

in which *n* is the number of spatial data points, *x* is a spatial data point, *X*(*x*) is the normalized validation value for *x*, and *Y*(*x*) is the normalized value for the model or ensemble tested.

We also conducted rank-order comparisons using Spearman’s ρ as an accuracy measure, which showed consistent results (section S5).

To allow statistical comparisons, we bootstrapped with 1000 runs for 10% of the data sets (AG carbon and water supply) or 100 datapoints (country validations) reporting the mean and SD across these bootstraps. We tested all accuracies within the same bootstrap run, allowing pairwise comparisons. We assessed accuracy differences with pairwise *t* tests (Matlab ttest-tool). The mean of pairwise differences per run is generally larger than the difference between the averaged accuracies as shown in Fig. 3. The pairwise combinations included median accuracy among models [i.e., indicating a random pick among models (*20*)], the median ensemble and the median ensembles of the other four service as proxies. Since we used the same statistical test five times per service per comparison, we used a Hochberg’s step-up correction (*49*) to account for multiple tests on the resulting average *P* values. Hochberg’s step-up correction is seen as more powerful than Sidak, Bonferroni, and Holms correction methods, which are known to underestimate true effects (*49*). A comparison with six other approaches to creating ensembles from (*20*) are reported in section S7.

### Spatial comparison of ensemble accuracy with development and equality per country

We explored possible drivers of the spatial variation of ES ensemble accuracy, testing if ensembles are more accurate in more economically developed countries with relatively higher levels of data, research, and model development. We used the following metrics:

• The HDI of 2019, as metric developed by the UN Development Programme being a summary measure of proxies for three important ends of development: access to health, education, and goods (*50*). Downloaded from hdr.undp.org/en/indicators/137506.

• The following World Development indicators were downloaded from The World Bank (databank.worldbank.org/home.aspx) using 2018 data (except GDP per capita) or the latest available entry before:

º GDP per capita downloaded from World Bank 2019 in U.S. dollar purchasing power parity, supplemented for missing countries by Central Intelligence Agency data for 2018 (cia.gov/the-world-factbook/field/real-gdp-per-capita/country-comparison).

º Income equality following the Gini index measuring the extent to which the distribution of income among households within an country deviates from an equal distribution.

º The number of researchers engaged in research and development (R&D), expressed as per million.

º Gross domestic expenditures on R&D, expressed as a percent of GDP.

After exporting all above outputs to Matlab v7.14.0.739, we correlated these metrics one by one (Metric) with the per-validation point accuracy of the median ensemble, calculated as the inverse of deviance per point $\left[D_{\left(x\right)}^{↓}=(1-|X_{\left(x\right)}-Y_{\left(x\right)}|)\right]$, using a SS-type I model with the Matlab Anovan tool:

$$D_{\left(x\right)}^{↓}∼\beta_{0}+\beta_{1}{\mathrm{Auto}}_{\left(x\right)}+\beta_{2}{\mathrm{Metric}}_{\left(x\right)}+\varepsilon$$ (2)

in which *D^↓^*_(*x*)_ is the accuracy for polygon *x*, with effect sizes β and error ε.

We incorporated a correction for potential spatial autocorrelation through inclusion of a covariate (Auto) before estimating the correlation of the metric of interest, describing relatedness between individual outputs in deviance with the Euclidean distances among centroids of polygons/points (*13*, *51*). We used a maximum spatial autocorrelation effect range of 5°. To equalize degrees of freedom across services and avoid high degrees of freedom inflation of *F* values for AG carbon and water supply, resulting in near-zero *P* values even for very weak effects, an iteration method was used taking a standard sample size of 178 datapoints (the minimum *N* across services). Not setting a default number of bootstraps, we used a convergence iterations method, stopping the iterations after the mean sum of squares of each factor over all iterations will not have changed by more than 0.05% with an extra iteration, consistently for 25 tries sequentially (see codes on github.com/GlobalEnsembles and https://doi.org/10.5281/zenodo.7687580). Furthermore, we explicitly test for potential higher accuracy in more economically developed countries using a one-sided *P* value distribution (two sided is reported in section S5). The presented *F* values themselves are mirrored accordingly to represent the one-sided significance distribution. Since, for each ES, all metrics and the interaction (Eq. 3) are calculated for the identical set of *D^↓^*_(*x*)_ per point and hence the spatial autocorrelation among those, we used a Hochberg’s step-up correction (*49*) of significance to account for the use of eight tests, as in step 5. Identical tests using Spearman’s ρ as accuracy measure are reported in section S5.

Since individual wealth may be better represented by the distribution of wealth around the mean (i.e., GDP per capita), we also ran Eq. 2 as a two factor interaction model for GDP per capita and income equality with type I sum of squares between spatial autocorrelation and the tested factors and type III among factors and interaction following

$$D_{\left(x\right)}∼\beta_{0}+\beta_{1}{\mathrm{Auto}}_{\left(x\right)}+\{\beta_{2}{\mathrm{Equity}}_{\left(x\right)}+\beta_{2}{\mathrm{Equality}}_{\left(x\right)}+\mathrm{Interaction}+\varepsilon \}$$ (3)

## Supplementary Materials

### This PDF file includes:

Supplementary Information SI-1 to SI-7 Tables S1 to S6 Figs. S1 to S16 References

## Notes

View/request a protocol for this paper from *Bio-protocol*.
