# Supplementary material for: Model ensembles of ecosystem services fill global certainty and capacity gaps
Source: Sci Adv. 2023 Apr 7;9(14):eadf5492. doi: 10.1126/sciadv.adf5492 (PMC10081842; doi:10.1126/sciadv.adf5492)
Supplement: Supplementary file 1 — Supplementary Information SI-1 to SI-7 Tables S1 to S6 Figs. S1 to S16 References [file sciadv.adf5492_sm.pdf]

Science Advances
AAAS

Supplementary Materials for

Model ensembles of ecosystem services fill global certainty and capacity gaps

Simon Willcock *et al.*

Corresponding author: Simon Willcock, simon.willcock@rothamsted.ac.uk

Sci. Adv. **9**, eadf5492 (2023)
DOI: 10.1126/sciadv.adf5492

This PDF file includes:

Supplementary Information
Tables S1 to S6
Figs. S1 to S16
References

SI-1. Input data and post processing for InVEST, TEEB, Scholes, ARIES and Co$ting Nature

Below, the input data used in InVEST, TEEB and the Scholes model are described, adapting descriptions from (*8*) with added global data sources.

S-1-1. InVEST

InVEST is a suite of stand-alone and free models from the Natural Capital Project (*24*) that are downloaded as one package from the website (https://naturalcapitalproject.stanford.edu/software/invest). Extended descriptions of each model are provided in the online user guide (https://storage.googleapis.com/releases.naturalcapitalproject.org/invest-userguide/latest/index.html#).

InVEST comprises of independent modules, each module covering one ecosystem service (ES). In this study we used three of the more widely used modules; the water yield module (*28*), the carbon module (*59*) and the recreation module (*60*) of release v3.8.9, which was the current version at the time of conducting this part of our research in 2021. Two of the InVEST modules (water and carbon) do not contain autonomously drawn-in data sources. Instead, all data sets need to be provided manually. The output generated is at the grain equal of the provided land cover map. In contrast the recreation module is based on drawn-in data, defined by entered years, gridcell size (spatial resolution) and area.

InVEST water yield module

The InVEST water yield model is a process model, built as a hydropower module, identifying quantitatively how much water or economic value each part of the landscape contributes to hydropower production. This is done by estimating water run-off through a single point. The model has three components: water yield, water consumption, and hydropower valuation. We used the first component here, using the gridded outputs of water run-off per gridcell, allowing standardising extraction per validation polygon among model outputs and ensembles. The required entered watersheds were dummies, over which InVEST would sum separately, and are not used for any of our outputs.

As input data we used:

- Summed monthly precipitation using WorldClim version 2.1 at a 0.008333° resolution: (worldclim.org/worldclim21.html).
- Summed monthly potential global potential Evapotranspiration in mm from CGIAR-CSI Global Aridity index v2 on a 0.008333° resolution: (cgiarcsi.community/2019arity).
- Root restricting raster was obtained from the Harmonized World Soil Database v1.2: webarchive.iiasa.ac.at/HWSD using the Reference soil depth. Subsequently, this polygon layer was converted to an exact 0.008333° grid.
- Plant Available Water Content (PAWC) raster was obtained from the Harmonized World Soil Database v1.2: webarchive.iiasa.ac.at/HWSD.
  PAWC was calculated from individuals soil factions ($S_{content}$) of sand, silt and clay for the upper layer (0-30 cm) and lower layer (>30 cm), following:
  $$PAWC = \sum_{content=sand}^{silt,clay} \left(S_{coontent} \times (FC_{content} - PWD_{content})\right) \qquad \text{Eqn. S1}$$
  With plant available water (PWD) and field capacity (FC) following (*61*), listed in Table S1.
  The two layers were then averaged, weighted on their depths with 30 cm and [root restricting depth – 30 cm]. Subsequently, this polygon layer was converted to an exact 0.008333° grid.
- Land cover followed MODIS MCD12Q1 v 5.1. land cover in 17 classes (*44*), resampled to a 0.008333° resolution based on dominant land cover.
- The maximum rooting depth and evapotranspiration coefficient (Kc), as look-up table per land cover class, were from (*8*).

The water yield module was run for 10 different seasonality factors (Z; Table S2). In postprocessing, each cell was assigned the most appropriate single output for the cell's number of rain days. Here, the Z factor was based on [Z= 0.2 ∗ N], where N is the average number of rain days per year (*28*). The number of rain days was calculated from summing the monthly days with rain per year from the FAO Wet Day Frequency per month (data.apps.fao.org/-map/raindays). Using rounded natural breaks on the number of rain days, the Z factors per cell were assigned following Table S2.

Table S1. PWD and FC factors for PAWC calculation from soil fractions following (*61*)

	PWD	FC
Sand	0.05	0.1
Silt	0.14	0.28
Clay	0.3	0.42

Table S2. Assignment of rain days per year ranges to InVEST seasonality (Z) factors.

Z used	FAO Rain days	
	Minimum	Maximum
3	0	35
10	>35	65
15	>65	90
21	>90	115
26	>115	140
30	>140	160
34	>160	180
39	>189	205
44	>205	235
55	>235	311

InVEST carbon module

This InVEST module is a look-up table based model, which uses maps of land cover and data on wood harvest rates, harvested product degradation rates, and stocks in four carbon pools (aboveground biomass, belowground biomass, soil, dead organic matter) to estimate the amount of carbon currently stored in a landscape or the amount of carbon sequestered over time. We did not employ the sequestration functions and restricted ourselves to the above ground, standing carbon pool only to match our validation sets. The model generates gridded maps of standing carbon per land cover based on the carbon pools at the grain equal of the provided land cover map (EU-Copernicus GLC 2000 land cover). This land cover map was used for the IPCC standing carbon assessment tables in combination with ecofloristic zones (*52*).

As input data we used:

- Land cover from the EU-Copernicus Global Land Cover 2000 Project (GLC 2000) with 23 classes at an original 0.008929° resolution: resampled to an exact 0.008333° grid. https://forobs.jrc.ec.europa.eu/products/glc2000/products.php
- Ecofloristic zones from CDIAC (https://cdiac.ess-dive.lbl.gov/epubs/ndp/global_carbon/carbon_documentation.html)
- Carbon stocks per land cover class per ecofloristic zone via CDIAC [IPCC Tier 1; (*52*); https://cdiac.ess-dive.lbl.gov/epubs/ndp/global_carbon/carbon_tables.pdf]. We only used above ground stored carbon values. In the model carbon stocks for the other layers, below ground, soil and dead material were set to 0.

This simulated above ground carbon in tonnes hectare^{-1} fully overlaps with the predictions from ARIES, performed with identical input data. To enhance the carbon stock estimate, the predicted values in forest ecosystems (IPCC Tier 1 categories 1:10 & 17) were multiplied with MODIS Vegetation Continuous Fields percent tree cover [MOD44B v6; (*62*)] providing a finer scaled map of potential carbon within forest ecosystems.

InVEST Recreation module

The InVEST recreation model predicts the spread of person-days of recreation. In the absence of empirical data on visitation, the model uses a proxy for visitation (geotagged photographs posted to the website flickr; see online user guide), which are automatically drawn while running the module. The module was run on a global 5° fishnet of land containing cells at a 0.0083333° resolution of square cells – i.e., the areas looped over are 5° squared polygons, rasterised to 0.083333° cells. The period chosen was 2016-2017 to keep within a manageable server usage time. The global area included all land area up to 60° North, supplemented by all of Iceland and Scandinavia. These approximately 750 individual 5° outputs were joined into one global map.

SI-1-2. TEEB: benefit transfer following (*45*)

Costanza *et al.* (*45*) provide estimates of the monetary value of global ES based on data from the TEEB study using benefits transfer (*63*). Benefit transfer estimates the value/quantity of ecosystem services using an aggregate total by assigning a constant unit value per area of ecosystem type. The assigned values are derived at the global-scale in which forest ecosystems are split into tropical and temperate/boreal regions.

Following (*8*), we associated the 23 GlobCover 2009 land cover classes at an original 0.008929° resolution (due.esrin.esa.int/files/p68/-GLOBCOVER2009) with benefit values (*45*) (Table S3). We matched the cover categories with the reported ecosystem values from 2011 (measured in 2007 US$ ha-1 yr-1) following Table S4. In addition, based on Ecofloristic zones from CDIAC (https://cdiac.ess-dive.lbl.gov/epubs/ndp/global_carbon/carbon_documentation.html), we split all wooded zones in Tropical and Temperate to match the distinction from (*45*), as well as added an in-between subtropical category assumed to be the average among both (not in Table S4 for length reasons, but in all cases calculated as [0.5 x Tropical + 0.5 x Temperate]). We resampled resulting grids to an exact 0.0083333° resolution.

Table S3. Associations of benefit categories of (*45*) with ES services used in this study

Ecosystems in this work	Value category of Costanza *et al.* (*45*)
Water supply	Water Supply
Recreation	Recreation
AG carbon	Climate Regulation
Fuelwood	Raw Materials
Forage production	Climate Regulation with a forage production mask (SI-3)

.

Table S4. Translation factors of GlobCover Land cover classes to $-value following (*45*).

Class	GlobCover Description (shortened)	Land cover categories from Costanza *et al.* (*45*) & calculation	Region	Climate Regulation	Water Supply	Raw Materials	Recrea- tion
11	Post-flooding or irrigated croplands	Cropland		410.7	400	219.2	82.2
14	Rainfed croplands	Cropland		410.7	400	219.2	82.2
20a	Cropland grassland & forest mosaic	(0.65 x Cropland) +	Tropical	611.1	255.2	155.7	205.6
20b		(0.35 x (Forest + Grassland) /2)	Temperate	280	283.9	172.6	226.9
30a	Grassland, forest & cropland mosaic	(0.35 x Cropland) +	Tropical	769	166.1	118	296.7
30b		(0.65 x (Forest + Grassland)/2)	Temperate	201.4	215.3	147.2	333.3
40a	Closed to open broadleaved evergreen and/or	Tropical Forest	Tropical	2044	27	84	867
40b	semi-deciduous forest	Temperate Forest	Temperate	152	191	181	989
50a	Closed broadleaved deciduous forest	Tropical Forest	Tropical	2044	27	84	867
50b		Temperate Forest	Temperate	152	191	181	989
60a	Open broadleaved deciduous forest	Tropical Forest	Tropical	2044	27	84	867
60b		Temperate Forest	Temperate	152	191	181	989
70a	Closed needle-leaved evergreen forest	Tropical Forest	Tropical	2044	27	84	867
70b		Temperate Forest	Temperate	152	191	181	989
90a	Open needle-leaved deciduous or evergreen forest	Tropical Forest	Tropical	2044	27	84	867
90b		Temperate Forest	Temperate	152	191	181	989
100a	Closed to open mixed broadleaved and needle-	Tropical Forest	Tropical	2044	27	84	867
100b	leaved forest	Temperate Forest	Temperate	152	191	181	989
110a	Mosaic forest, shrubland & grassland	0.65 x Forest + 0.35 x Grassland	Tropical	1240.4	37.2	69.3	529.3
110b			Temperate	105.2	135.6	127.5	602.5
120a	Mosaic grassland, forest & shrubland	0.65 x Grassland + 0.35 x Forest	Tropical	739.4	45.5	61.8	319.1
120b			Temperate	77.2	102.9	95.8	361.8
130a	Closed to open shrubland	assumed 6% of Tropical value	Tropical	122.6	1.62	5.04	52.0
130b			Temperate	9.12	11.5	10.9	59.3
140	Closed to open grassland	Grassland		40	60	54	26
150	Sparse vegetation	n/a		0	0	0	0
160	Closed broadleaved forest regularly flooded, fresh water	Swamps/Floodplains		65	1217	358	2193
170	Closed forest regularly flooded, saline water	Tidal Marsh/Mangroves		488	408	539	2211
180	Closed to open grassland or shrubland or woody on regularly flooded	Swamps/Floodplains		65	1217	358	2193
190	Artificial surfaces and associated areas	Urban		904.7	0	0	5740
200	Bare areas	n/a		0	0	0	0
210	Water bodies	Lakes/Rivers		0	1808	0	2166
220	Permanent Snow and Ice	Ice/Rock		0	0	0	0
230	No data	n/a		0	0	0	0

SI-1-3. Photosynthetic capacity extended Scholes model

The Scholes model (*8*, *54*) to estimate grazing density is a carrying capacity model, which calculates the service of forage production potential. As an intermediate product, the model calculates a water supply layer as number of growth days (GD below), which we use as one of our water supply models. Below, we summarise the model and our extension to make it a global model.

This model output is intended to show land areas in terms of their forage production potential and not necessarily as a guide to economically viable grazing capacities (*8*, *54*). It is based on the potential of the vegetation to feed animals locally and does not include any transport of resources to other areas. The model was developed for South Africa by (*54*) and has been re-interpreted in (*8*). We refer to the full model description of (*8*). Here, we extend the model by adding photosynthetic possibility by including relative monthly solar radiation.

The Scholes model used in this study follows the re-interpretation of (*8*), necessary due to some lack of clarity in the original model description (*54*). In this interpretation the model is a hierarchical correction model per gridcell of the potential Livestock Units (LSU) in a gridcell:

$$potential\ LSU = \alpha \left\{ D_x \left(C_x \left\{ \left(A_x \left(log_{10} \left(GD_x^{\boldsymbol{Extended}} \times \frac{1}{\beta} \right) \right) \right) - B_x \right\} \right) \right\} \qquad \text{Eqn. S2}$$

With α a scaling parameter (set to 1), β = 0.24, x = a gridcell, A_x, B_x soil fertility parameters per gridcell, C_x slope parameter per cell, D_x land cover parameter per cell, GD_x growth days (Eqn. S3). *Extended* denotes the proxy of photosynthetically active radiation.

1. The first innermost level $\left(log_{10} \left(GD_x^{\boldsymbol{Extended}} \times \frac{1}{\beta} \right) \right)$ describes the intrinsic maximum forage potential capacity for a given climate, estimated by the annual number of days that rainfall exceeds evapotranspiration. This term is described as 'Growth Days' and is the Scholes water supply model in this study.

   The principal control on rangeland forage production is the soil water balance. The productivity of grasses, shrubs and trees is strongly correlated with the quantity of water, which they transpire relative to the quantity which they could potentially transpire if the soil moisture supply was unlimited. This is calculated as the number of annual days rainfall exceeds evapotranspiration and is used as water supply model in this work.

   The annual number of days rainfall exceeds evapotranspiration was calculated in monthly bins, this calculation is used as Scholes water supply model:

$$GD_x = \sum_{m=1}^{12} \left(\frac{d_m * P_{(m,x)}}{E_{(m,x)}} \right) \qquad \text{Eqn. S3}$$

   With P = precipitation in gridcell x in month m; E = potential evapotranspiration in gridcell x in month m; m = month (1 to 12), x = 1 km^2 gridcell and d = number of days per month.

   We used the follow source data:

   - Monthly precipitation using WorldClim version 2.1. on a 0.008333° resolution (worldclim.org/worldclim21.html).
   - Monthly potential global potential Evapotranspiration in mm from CGIAR-CSI Global Aridity index v2 on a 0.008333° resolution (cgiarcsi.community/2019arity).

   The original model is focused on South Africa, developed at Stellenbosch (South Africa, at -33.93 South, 18.86 East), assuming a default photosynthetic period and solar quantity. To extend this

model to a global scale, this assumption needs to be altered. To do so, we added a further correction element to the main equation based on relative solar radiation quantity in monthly summed watts hour^{-1} m^{-2} as proxy of photosynthetically active radiation.

We used the SRTM 90m Digital Elevation Database v4.1. as source data, resampled to a 0.008333° resolution based on the mean elevation. From this DEM, we calculated the monthly solar sum per gridcell (Sol_x) using the ArcGIS *area solar radiation tool*, which was made relative to the annual solar estimate in Stellenbosch.

Following GD was corrected as: $GD_{(m,x)}^{corrected} = \left(GD_x \times \left(\frac{Sol_x}{Sol_S}\right)\right) \times \tau$ Eqn. S4

With Sol_x, the monthly solar total for gridcell *x*, $GD_{(m,x)} = \left(\frac{d_m * P_{(m,x)}}{E_{(m,x)}}\right)$, see above, Sol_S equals the monthly total at Stellenbosch (*i.e.,* this makes it relative to the original location). τ is the Stellenbosch correction factor which is the ratio of annual summed solar radiation at Stellenbosch compared to the annual maximum globally: τ equals 1.278.

2. The second level (donated with {} brackets) calculates the intrinsic forage production capacity corrected for soil conditions [*A* & *B*; see (*8*) for explanation and tables]. Soil type data used followed the FAO references soil groups (RSGs) of the Harmonized World Soil Database v1.2 (webarchive.iiasa.ac.at/HWSD) with fertility assessment in three categories; high-medium-low) following FAO descriptions (fao.org/3/a-a0510e.pdf and fao.org/fileadmin/-pdf_documents/wrb2007_red.pdf).

3. The third level provides a correction for slope (*C*). Note that, being a correction model, *C* equals 1 in conditions not limited by slope. It is estimated no domesticated grazing animals are present above 35° degrees slopes (≈ 70%). The slope correction was calculated as:

$$If \begin{cases} S_x \le 4°: C_x = 1 \\ S_x > 4° \& \le 35°: C_x = (-\gamma \times S_x) + \theta \\ S_x > 35°: C_x = 0 \end{cases} \qquad \text{Eqn.S5}$$

With S_x is slope in degrees, γ = 0.0323 and θ = 1.129.

The slope was calculated using ArcGIS *Surface-slope* tool based on the SRTM 90m Digital Elevation Database v4.1, resampled to 250 meters (cgiarcsi.community/data/srtm-90m). Subsequently the resulting *C*-factor was averaged to an exact 0.0083333° resolution.

4. The fourth level provides a correction for land cover (*D,* again with {} brackets). Land cover has a large effect on the potential forage production; if covered with other vegetation types proportionally less forage production potential is present (*54*). For this correction layer we used MODIS MCD12Q1 v5.1. land cover in 17 classes (*44*), resampled based on dominance to a 0.008333° resolution. Since the original calculation in (*54*) was done on South African land cover maps, (*8*) made an interpretation for which Scholes class was most applicable per MODIS cover class, which we extended to non-binary here (see Table S6). Note that being a correction model, D equals 1 in default non-improved grass- and herblands.

5. For the parameters, *α* equals 1 . β is the transformation coefficient [page 6 in (*54*)] from number of growth days to mean annual precipitation, and is set to 0.24. Note that β was not included in our calculation of growth days as water supply model (Eqn. S3).

SI-1-4. Recreation and additional post-processing for ARIES and Co$ting Nature
Recreation is an ensemble with multiple different modelling methods. Two approaches are based on observations (Photo uploads: InVEST and Co$ting Nature), two approaches are based on human population movement through gravity functions (ARIES and Chaplin-Kramer), a third approach is the benefit transfer approach following TEEB.

All approaches predict recreational pressure, so are comparable, but contain a whole different set of assumptions, units and resulting output. Photo approaches are heavily biased to Europe, North America, and very localized inbound tourism hotspots, showing observed (proven) recreation potential. However, photo approaches largely fail to capture domestic recreation in most of the developing world, where photo uploads are less common. In addition, radiating gravity functions provide potential values for domestic tourism, assuming equal tourism opportunities and quality of infrastructure throughout (although this is partly corrected for in ARIES; see below). Benefit transfer approaches (i.e. TEEB) share a similar assumption (i.e. no variation with each land cover). Combining models with different assumptions, and therefore biases, likely maximises the portfolio effect (see Discussion). For example, the photo approaches strongly complement the population based gravity functions of potential recreational value; e.g. tourism in national parks in countries such as Kenya, Tanzania and South Africa are visible within the ensemble maps (Figure S7) to a much higher degree than would be expected if calculated from population density alone. This is also apparent in Europe, where next to cities, mountain ranges highlight as top recreational spots (Figure S8).

Note: The photo approach may be biased in urban areas (i.e. where the number of photos which have been taken in those regions may not indicate the number of people that are recreating in the open landscape enjoying the nature). We opted not to mask out urban settlements as by making the full coverage maps available, we provide the opportunity to the user to mask any area based on the research questions asked.

However, we call for the development additional recreation models. Because only five recreation models were available, the median ensemble shows sudden spatial transitions – e.g. estimates driven by continuous gravity models shifting to being more influenced by photo approaches in areas with low population densities (most apparent in South-America at the edge of the Amazon basin, Figure S8).

Co$ting Nature postprocessing
Co$ting Nature was run in 10°-tiles that are each individually normalised by the framework. This implies that each tile contains the maximum value of 1 and all other gridcells are relative to that, independent of values in other tiles. Therefore tiles cannot be put together in a simple mosaic, but need recalibration to a common source that scales among tiles. For example, a Co$ting Nature carbon value of 1 in a certain tile might correspond to 50 tonnes, whereas in the neighbouring tile the value of 1 could correspond to 100 tonnes. In this two tile example, the first needs to be rescaled to 0.5 before the tiles can be combined.

We used normalised global-scale outputs from other models as comparators for rescaling Co$ting Nature. Creating a ArcGIS *fishnet* of 10°-tiles, the single maximum value within the tile location was taken from the comparator model output. Subsequently, this single value was multiplied with all Co$ting Nature values within the tile. For example, if the maximum of the comparison model for a certain 10°-tile was 0.76, all values of Co$ting Nature for that certain tile would be multiplied by 0.76. This would not alter the relative rankings of Co$ting Nature within tiles, but rescales all tiles to a common global source. By using a single maximum value per tile of the comparator model output, some roughness at edges is unavoidable but the additional spatial autocorrelation between these model outputs remains as limited as possible. To remove outliers, the comparators were normalised with a one-sided 95% winsorising protocol following (*20*) prior the *fishnet* procedure.

In detail per service:

- Co$ting Nature water supply is not rescaled, as the model output is in physical units (m^3) of run-off per gridcell (i.e. not accumulated flow).
- Co$ting Nature carbon was rescaled per 10°-tile against the globally normalised maximum value of a mean 4-way ensemble of the outputs of InVEST, ARIES, Global Forest Watch and Conservation International (Table 2). These models each provide full coverage of either biomass or carbon – which are identical in normalised form – at similar scales and without land cover masks applied for non-forest areas.
- Co$ting Nature recreation was set as the sum of culture-based tourism and nature-based tourism photo index values (both ranged 0-1). These two Co$ting Nature outputs are generally mutually exclusive per grid cell. The few values above 1 were trimmed. Thereafter, the resulting combined output was rescaled per 10°-tile against the globally normalised maximum value of the InVEST recreation output, being a similar photo approach.
- Co$ting nature fuelwood was rescaled per 10°-tile against the globally normalised maximum value of a mean 3-way ensemble of the outputs of InVEST, ARIES, and Conservation International (Table 2), all after woody mask appliance. These models each provide full coverage at similar scales.
- Co$ting nature fodder production was rescaled per 10°-tile against the globally normalised maximum value of Gilbert *et al.* (*43*) (Table 2), which is providing Livestock Units.

Since we use the in-built input data for Co$ting Nature, we minimised the time span of the independent model runs to reduce the likelihood of input updates. The majority (190) of Co$ting Nature tiles were downloaded between 7th December 2020 and 29th January 2021, with 6 tile replacements of compromised zipped files downloaded on 2nd February 2021. As first assessment of feasibility, 10 European tiles were downloaded on 9th and 10th November 2020. The (paid) license provides access to all policysupport.org systems (Co$tingNature, WaterWorld) for application anywhere globally at 1km or 1ha spatial resolution. Our approach ensures that practitioners with resources to obtain the same license can replicate our methods.

ARIES recreation

For ARIES recreation, the access to nature predictions were run on a per country basis, with predictions indicating numbers of people normalised within countries –*i.e.*, all ranged from 0-1 within countries. Assuming that the value of recreation is proportional to the wealth of countries, we multiplied with GDP per head per country [GDP per head x modelled value]. Which, after subsequent global normalisation (see below), provides a worldwide scaling of these per country values.

SI-2. Converting grazing animals numbers to LSUs

For gridded livestock predictions (*43*) we combined available livestock classes – buffalos, cattle, chickens, ducks, horses, goats, pigs & sheep – by converting their numbers per gridcell to FAO livestock units (LSU) following (*64*), split into tropical and non-tropical categories. This method is based on potential grazing biomass per animal compared to a fully-grown cow. Subsequently the LSU's were summed per gridcell. A list of conversion factors per livestock type is provided in Table S5.

Table S5. Livestock numbers to LSU Units conversion factors

Livestock Type	Tropical	Non-Tropical
Cattle	0.7[†]	1[‡]
Horses	0.8[†]	1[‡]
Sheep	0.1[†]	0.08‡
Buffalos	0.7[‡]	
Chickens	0.01[†]	
Ducks	0.01[§]	
Goats	0.1[†,‡]	
Pigs	0.2[†,¶]	

†FAO (2011;2018); ‡Chesterton (2006); § assumed as Chickens; ¶Nix (2009);

SI-3. Forage production and fuelwood masks for standing carbon

Models that predict potential fuelwood or forage production are rare; Co$ting Nature being a notable exception by independently calculating both. Thus, following and extending (*8*), we built predictors using the outputs from applicable carbon models, with the requirement that they cover all ecosystems and not only forest, including mixed vegetation categories (Table S6). The rationale is that the supply of these two ES are directly dependent on the amount of biomass present, which is also what underpins estimates of stored vegetation carbon. We did so by using MODIS-based land cover [MCD12Q1 v 5.1.; (*44*)] masks for grassland vegetation for seven out of the 12 employed forage production models (see Table 2 main text) and woodland vegetation for seven out of nine fuelwood models. Due to the subsequent normalisation (see main text), there is no requirement to use any transition factors, since any constant will drop out with normalisation [such the 12.7% factor following (*65*)].

- For forage production, we applied a spatial mask to derive predominantly grassland carbon from standing carbon outputs. We excluded areas in which little to no forage production was expected (0 values in Table S6) and included areas in which most of the above-ground carbon is assumed to be available for forage production (positive values in Table S6). The resulting carbon layer was considered as available for forage production, equivalent to the layer of LPJ-GUESS's C3 and C4 grasses combined. In contrast to (*8*) we did not use a binary system but employed the Scholes grazing model land cover correction factors, correcting for areas in which less vegetation is present [see (*8*)].
- For fuelwood we used a spatial mask to derive woody carbon from standing carbon outputs in land cover categories that have a substantial amount of woody vegetation (Table S6). Therefore, this mask includes forest areas and closed shrublands. In tropical areas, we assumed that open shrublands and woody savannas could be used for fuelwood collection but with reduced per area

factors compared to forest land cover, correcting for less woody vegetation present. For this we split the MODIS raster with the IPCC carbon zones (*36*), distinguishing between tropical carbon versus not tropical category zones. Similarly, we have included the Cropland/Natural Vegetation Mosaic with a partial per area ratio.

Table S6. Spatial mask with product factor for land cover MODIS categories [MCD12Q1 v 5.1.; (*44*)]. Calculation followed per gridcell as: [cell value x factor], subsequently cells with factor 0 below were set to no-data to exclude untrue zeros when averaging across a validation polygon.

**MODIS (v 5.1.) Land cover class**	**Scholes correction factor for forage**	**Woody vegetation factor for fuelwood**	
		Tropical	Not Tropical
Water	0	0	0
Evergreen Needle leaf Forest	0	1	1
Evergreen Broadleaf Forest	0	1	1
Deciduous Needle leaf Forest	0	1	1
Deciduous Broadleaf Forest	0	1	1
Mixed Forests	0	1	1
Closed Shrublands	1	1†	1†
Open Shrublands	1	0.5†	0§
Woody Savannas	0.6	0.4‡	0§
Savannas	1	0	0
Grasslands	1	0	0
Permanent Wetland	0	0	0
Croplands	0	0	0
Urban and Built Up	0	0	0
Cropland/Natural Vegetation Mosaic	0.5	0.5‡	0.5‡
Snow and Ice	0	0	0
Barren or Sparsely Vegetated	0.3	0	0

†Assumed factors; ‡calculated as [1-grazing factor]; §assumed to be generally absent.

SI-4. Maps of validation data

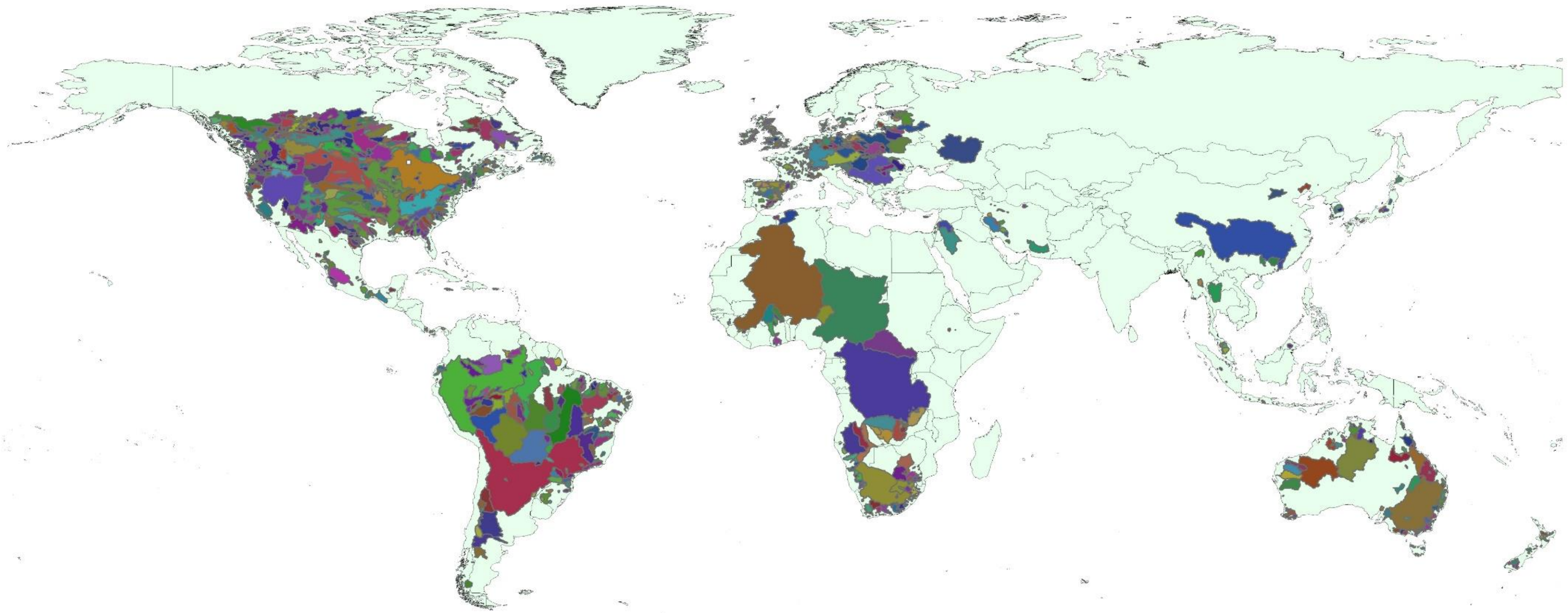

Figure S1. The watersheds generated for this study, being characterised by the location of GRDC weirs (N = 3746). The GRDC data (water flow in mean m^3 sec^{-1}) within each watershed provides validation data for water supply models. Partial overlap of watersheds is dealt with both at the point of output extractions and statistically through spatial autocorrelation correction.

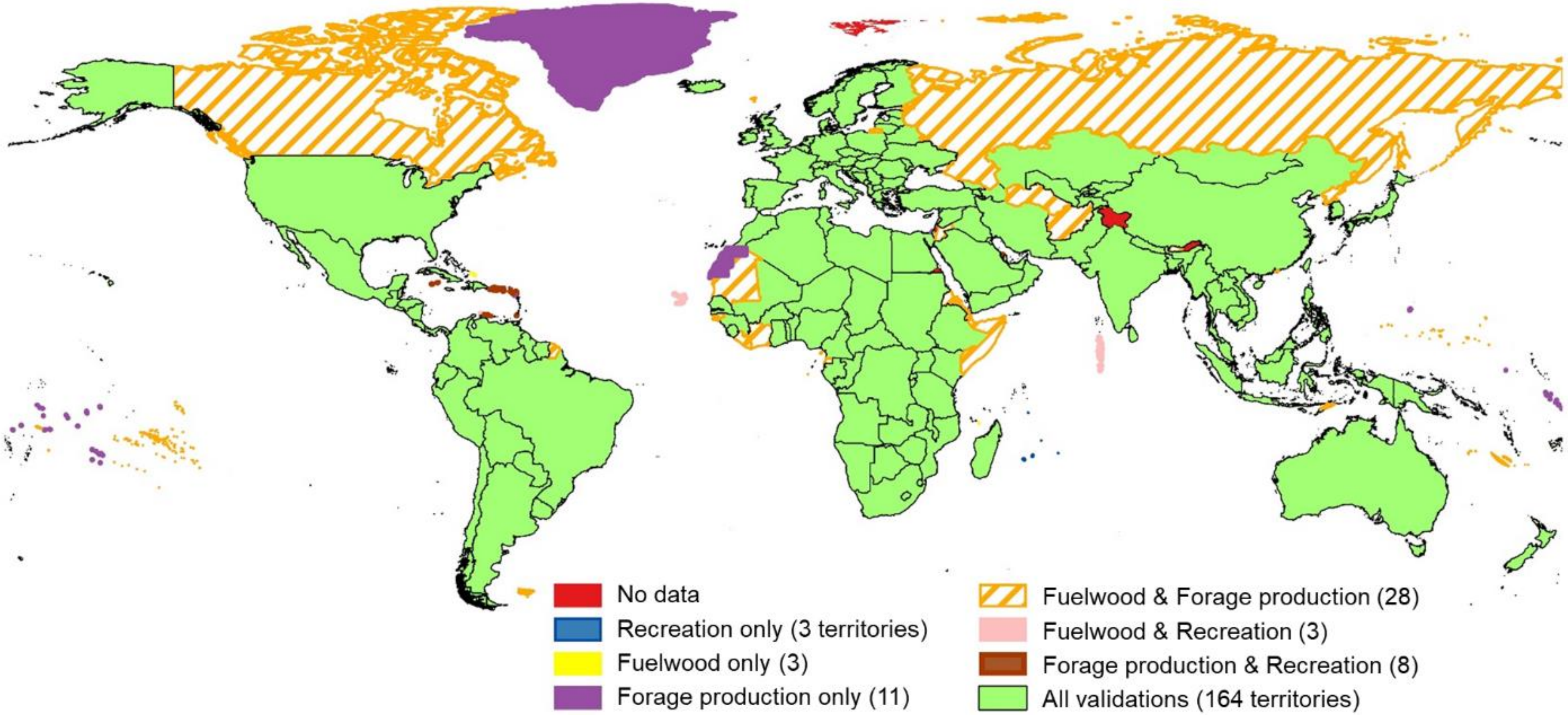

Figure S2. Depiction of which ecosystem service validation data are available per country and are included in the validation analyses (N with at least one service = 218). This uses WTCC Country sheets for recreation (2019 data, N = 178) and FAOStat, Fuelwood per country (2019 data, N= 195) and forage production per country (2018 data, N = 208). The map includes separate polygons for overseas territories. Countries are based on the GAUL-2 2014 definition. In cases where an overseas territories is represented separately in one of the validation data sets it was extracted as separate data point. We refer to all as 'countries', although we are aware not all have that status.

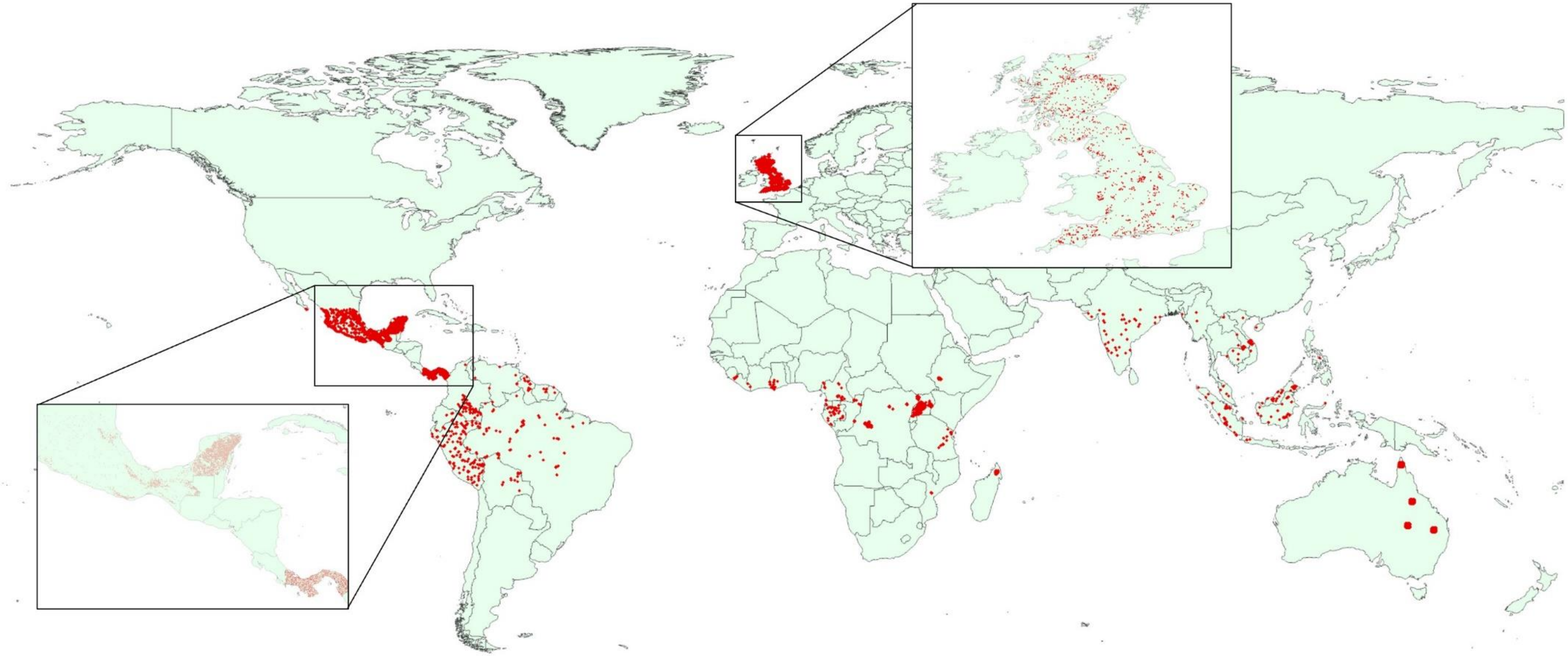

Figure S3. Location of the validation plots for pan-tropical biomass in forest plots belonging to the reference data of (*38*) (N = 14,478) and the centroids from carbon estimates in UK forest estates taken from (*20*) (N = 1,606), which are combined in this paper. Inset enlargements are added to indicate densities. Statistical calculations include spatial autocorrelation to remove the effects of the clustered data.

SI-5. Extended Results

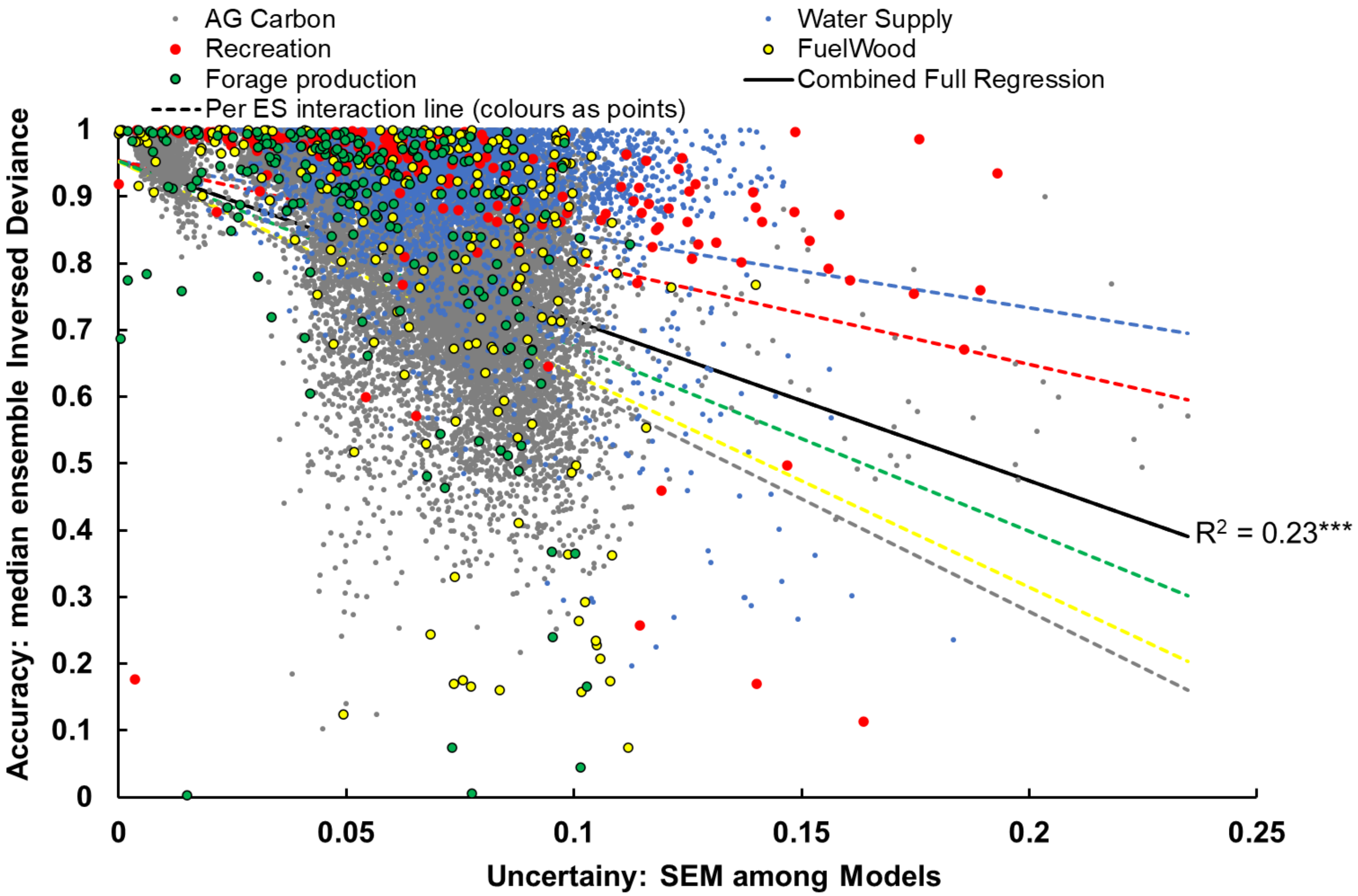

Figure S4. Standard error of the mean (SEM) of individual models correlates with deviance of the median ensemble from validation data (P<0.001). This is consistent across AG carbon (R^2 for single service: 0.28; P <0.001; grey); fuelwood (0.18; P <0.001; yellow), forage production (0.13; P <0.001; green), recreation (0.20; ; P <0.001; red), and water (0.06; P <0.05; blue) ecosystem services.

Table S7. Two-tailed correlations as F-values with (effect size) and significance of inverse of deviance per validation datapoint – where increasing accuracy is represented by increasing inverse of the deviance. This analyses extends Table 2 (main text), testing for potential changed accuracy of the ensembles against characteristics indicating the research capability of countries for five services. Accuracy improvement is the mean of pairwise comparisons for 1000 bootstraps. We use a standardised maximum DF of 178 among services following a bootstrap convergence model where as many bootstrap are taken until mean SS for all factors among runs becomes fixed. Significance of the presented F-values were assessed taking account of multiple tests, using Hochberg's step-up correction with 8 tests per ES, which are interdependent because of sharing the same spatial autocorrelation term, all DF = 1. Characteristics are calculated independently as [Y ~ Spatial Autocorrelation + Characteristic + error], with Spatial Autocorrelation to a maximum of 5° between country centroids. An interaction model is added testing for interactions between GDP and income equality as [Y ~ Spatial Autocorrelation + GDP per capita + income equality + Interaction + error] with type III Sum of Squares. Water supply is per bespoke watershed, recreation, fuelwood and forage production per country.

	**Water Supply**	**Recreation**	**AG Carbon**	**Fuelwood Production**	**Forage Production**
**Accuracy Improvement (deviance)**					
Ensemble vs. a random selected model (median among models	14%	6.1%	6.1%	3.4%	2.7%
**Spatial Autocorrelation**	15.3***	14.6***	211***	0.47	0.14
**Development/Equality per country**					
GDP per capita	1.38 (0.01)	34.2 (-0.09)***	1.21 (≈0)	3.58 (0.05)	0.24 (≈0)
Human Development Index	1.51 (0.04)	32.2 (-0.30)***	1.14 (≈0)	6.43 (0.26)*	0.25 (0.03)
Income Equality (Gini index)	1.01 (-0.03)	6.69 (0.26)*	1.37 (0.02)	10.6 (-0.64)*	0.28 (-0.06)
% People in R & D	1.44 (0.05)	22.0 (-0.52)***	1.09 (-0.01)	4.85 (0.49)	1.49 (-0.22)
% GDP to R & D	1.54 (-0.04)	9.77 (-0.57)**	1.10 (-0.02)	3.79 (0.72)	0.55 (-0.20)
**Interaction model**					
GDP per capita	1.76 (0.12)	0.96 (-0.16)	1.04 (-0.01)	0.11 (-0.04)	2.71 (-0.40)
Income Equality	1.67 (0.69)	0.22 (-0.48)	1.03 (-0.07)	0.42 (-1.06)	2.77 (-2.55)
GDP x Income Equality	1.67	0.34	1.04	0.16	2.67

* P < 0.05 corrected, ** P <0.01 corrected *** P <0.001 corrected

Table S8. One-tailed correlations as F-values with (effect size) and significance of inverse of the relative ranking difference per validation datapoint ($\rho^{\downarrow}_{(x)}$) ; the input to Spearman ρ). One-tailed tests were applied to test the hypothesis that the accuracy increases with higher values of each development/equality metric (two-tailed is presented in Table S9, including effect sizes). Whereas overall Spearman ρ is calculated using the Matlab standard function *corr*, the per validation point inverse of the ranking difference was calculated manually. This was done as:

$\rho^{\downarrow}_{(x)} = 1 - \left(\frac{\rho_{(x)}}{max(\rho)}\right), with\ \rho_{(x)} = \left(\frac{|Rank_{validator} - Rank_{ensemble}|}{Expected\ difference}\right)$, *Rank* the sorted ranks of the validator and ensemble of the selected data point x. *Expected difference* under a random correlation equals a $\frac{n}{3}$ ranking difference. $\rho^{\downarrow}_{(x)}$, thus includes normalisation against the maximum value among all points. See Table S7 for further statistical details.

	Water Supply	Recreation	AG Carbon	Fuelwood Production	Forage Production
**Accuracy Improvement (ρ)**					
Ensemble vs. a random selected model (median among models	51%	37%	29%	1.2%	22%
**Spatial Autocorrelation†**	11.5***	1.87	131***	10.2**	8.17**
**Development/Equality per country**					
GDP per capita	1.06	0.67	0.91	6.08	0.51
Human Development Index	1.19	0.62	0.79	11.2**	1.11
Income Equality (Gini index)	1.11	<0.01	1.48	<0.01	<0.01
% People in R & D	0.14	0.90	1.22	3.67	0.17
% GDP to R & D	0.07	1.20	1.24	3.39	0.16
**Interaction model**					
GDP per capita	0.07	0.01	0.64	0.14	<0.01
Income Equality	0.08	0.02	0.62	1.20	<0.01
GDP x Income Equality	1.52	2.44	0.31	0.08	8.48* (+)

†Two sided tested; * P < 0.05 corrected, ** P< 0.01 corrected; *** P <0.001 corrected.

Table S9. Two-tailed correlations as F-values with (effect size) and significance of inverse of the relative ranking difference per validation datapoint ($\rho^{\downarrow}_{(x)}$), testing for potential changed accuracy of the ensembles against characteristics indicating development & equality of countries for five services. See Table S7 for statistical details and SI-5-2 for ranking difference calculations.

	Water Supply	Recreation	AG Carbon	Fuelwood Production	Forage Production
**Accuracy Improvement (ρ)** Ensemble vs. a random selected model (median among models	51%	37%	29%	1.2%	22%
**Spatial Autocorrelation**	11.5***	1.87	131***	10.2**	8.17**
**Development/Equality per country**					
GDP per capita	1.06 (≈0)	0.3 (-0.01)	0.91 (0.01)	6.08 (0.08)*	0.51 (0.02)
Human Development Index	1.19 (0.01)	0.33 (-0.05)	0.79 (0.02)	11.2 (0.38)**	1.11 (0.1)
Income Equality (Gini index)	1.11 (0.06)	<0.01 (≈0)	1.48 (0.03)	6.26 (-0.55)*	5.88 (-0.51)*
% People in R & D	1.1 (-0.03)	0.2 (-0.08)	1.22 (0.02)	3.67 (0.47)	0.17 (0.01)
% GDP to R & D	1.61 (-0.10)	0.12 (-0.10)	1.24 (0.03)	3.39 (0.77)	0.16 (0.03)
**Interaction model**					
GDP per capita	1.55 (-0.06)	0.01 (0.03)	0.64 (0.05)	0.14 (0.08)	8.35 (-0.94)*
Income Equality	1.5 (-0.39)	0.02 (0.22)	0.62 (0.30)	0.12 (-0.41)	9.81 (-6.35)*
GDP x Income Equality	1.52	0.02	0.65	0.08	8.48*

* P < 0.05 corrected, ** P< 0.01 corrected; *** P <0.001 corrected

SI-6. Example case study regions

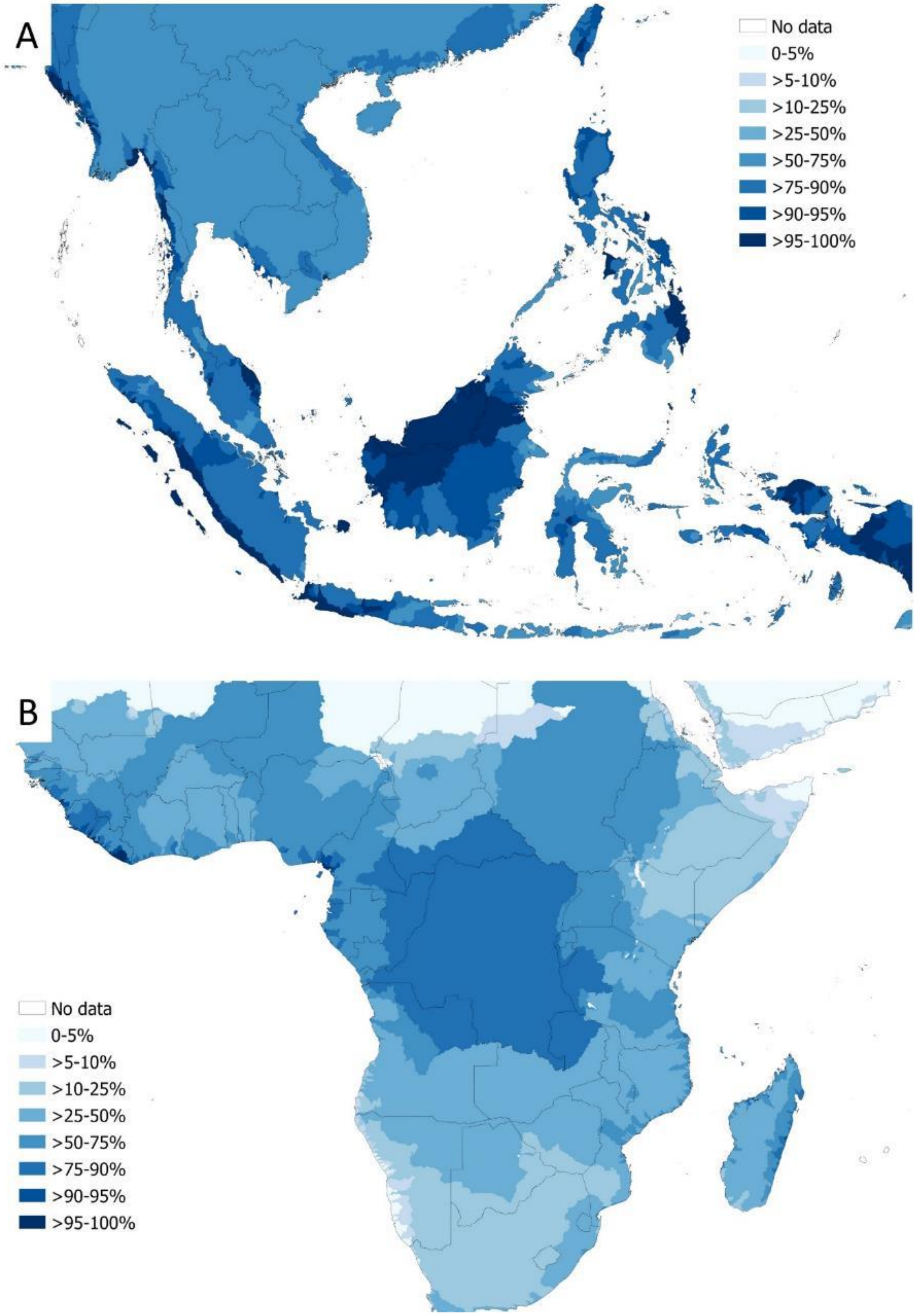

Figure S5. Example case studies for (A) South-Asia (scale 1:18.75M) and (B) Sub-Saharan Africa (1:30M) for **water**. Water is shown as accumulated flow estimate per catchment following the global HydroSHEDS catchments definition (*46*). True zero values (coloured) are distinguished from no-data (white).

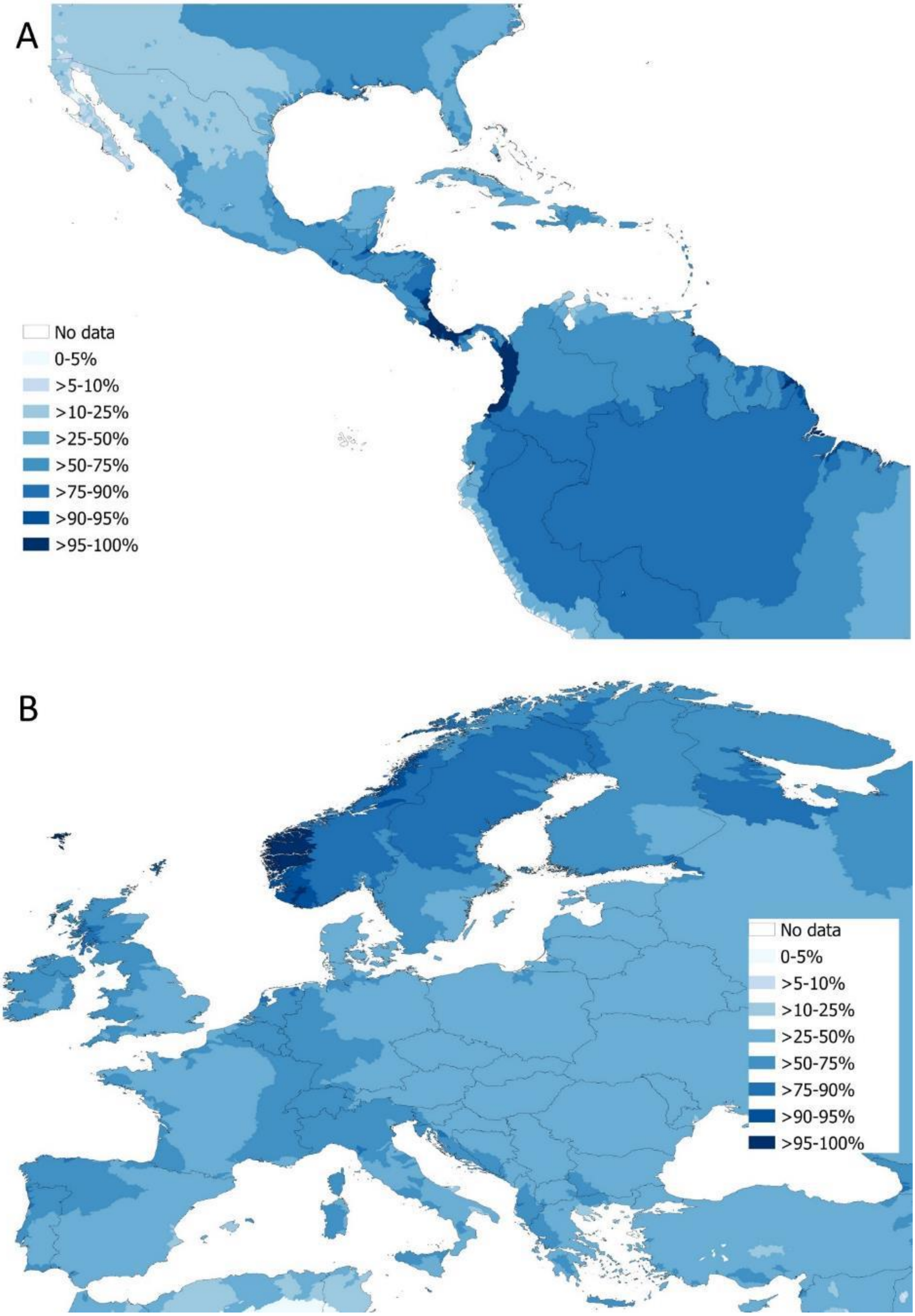

Figure S6. Example case studies for (A) Latin America (scale 1:30M) and (B) Europe (1:20M) for **water**. Water is shown as accumulated flow estimate per catchment following the global HydroSHEDS catchments definition (*46*). True zero values (coloured) are distinguished from no-data (white).

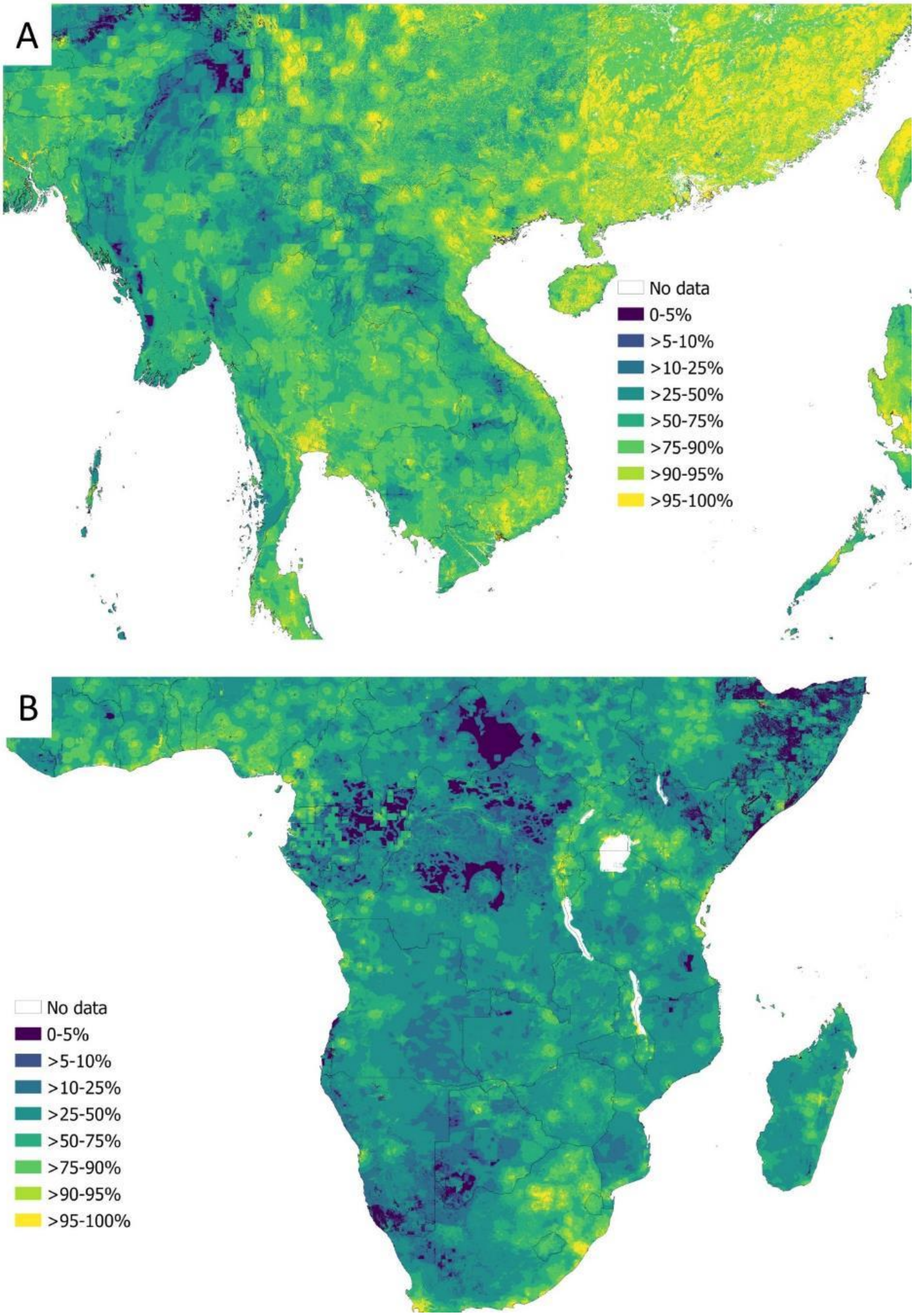

Figure S7. Example case studies for (A) South-Asia (scale 1:12M) and (B) Sub-Saharan Africa (1:25M) for **recreation**. True zero values (coloured) are distinguished from no-data (white).

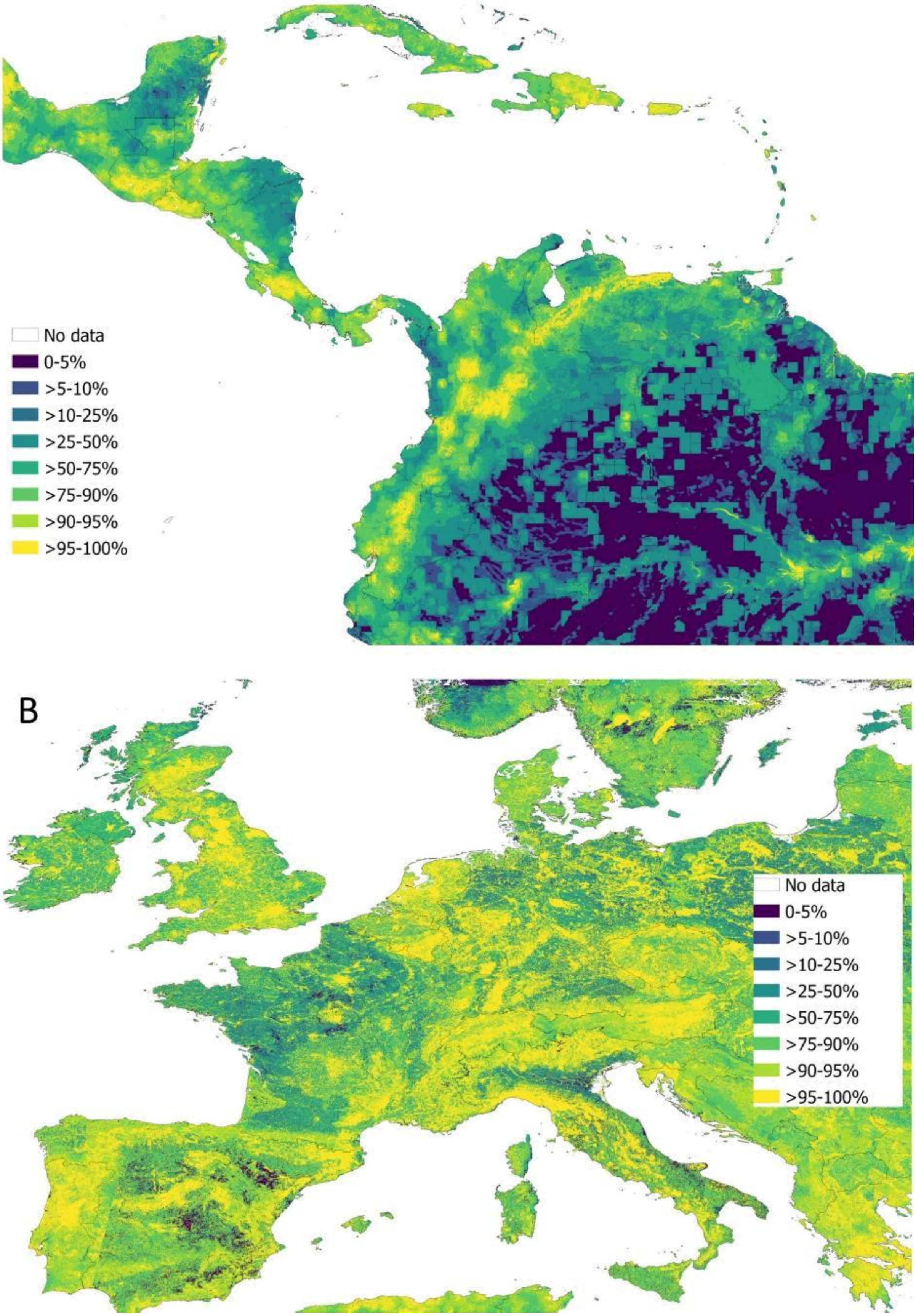

Figure S8. Example case studies for (A) Latin America (scale 1:16M) and (B) Europe (1:13M) for **recreation**. True zero values (coloured) are distinguished from no-data (white).

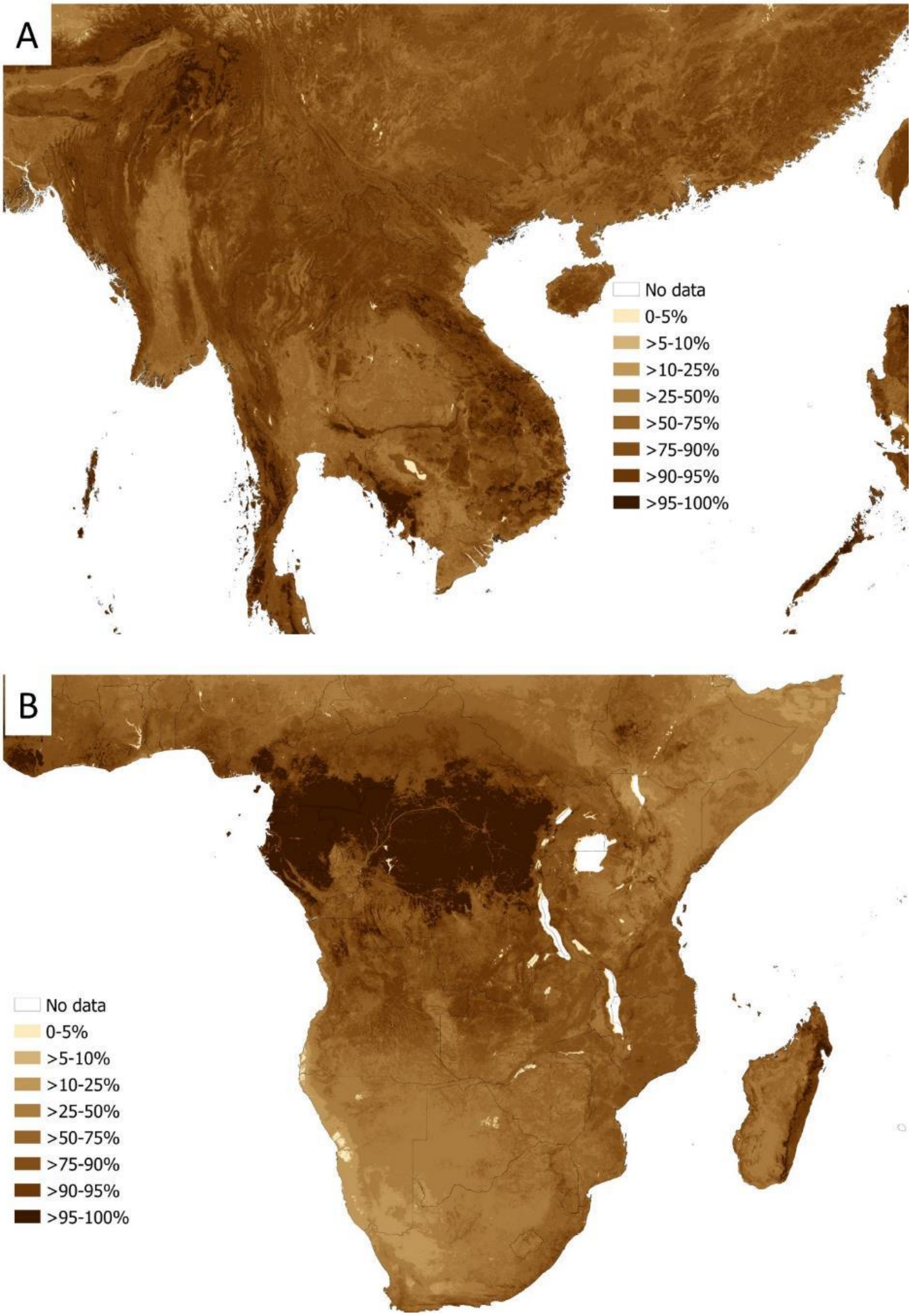

Figure S9. Example case studies for (A) South-Asia (scale 1:12M) and (B) Sub-Saharan Africa (1:25M) for **above ground carbon**. True zero values (coloured) are distinguished from no-data (white).

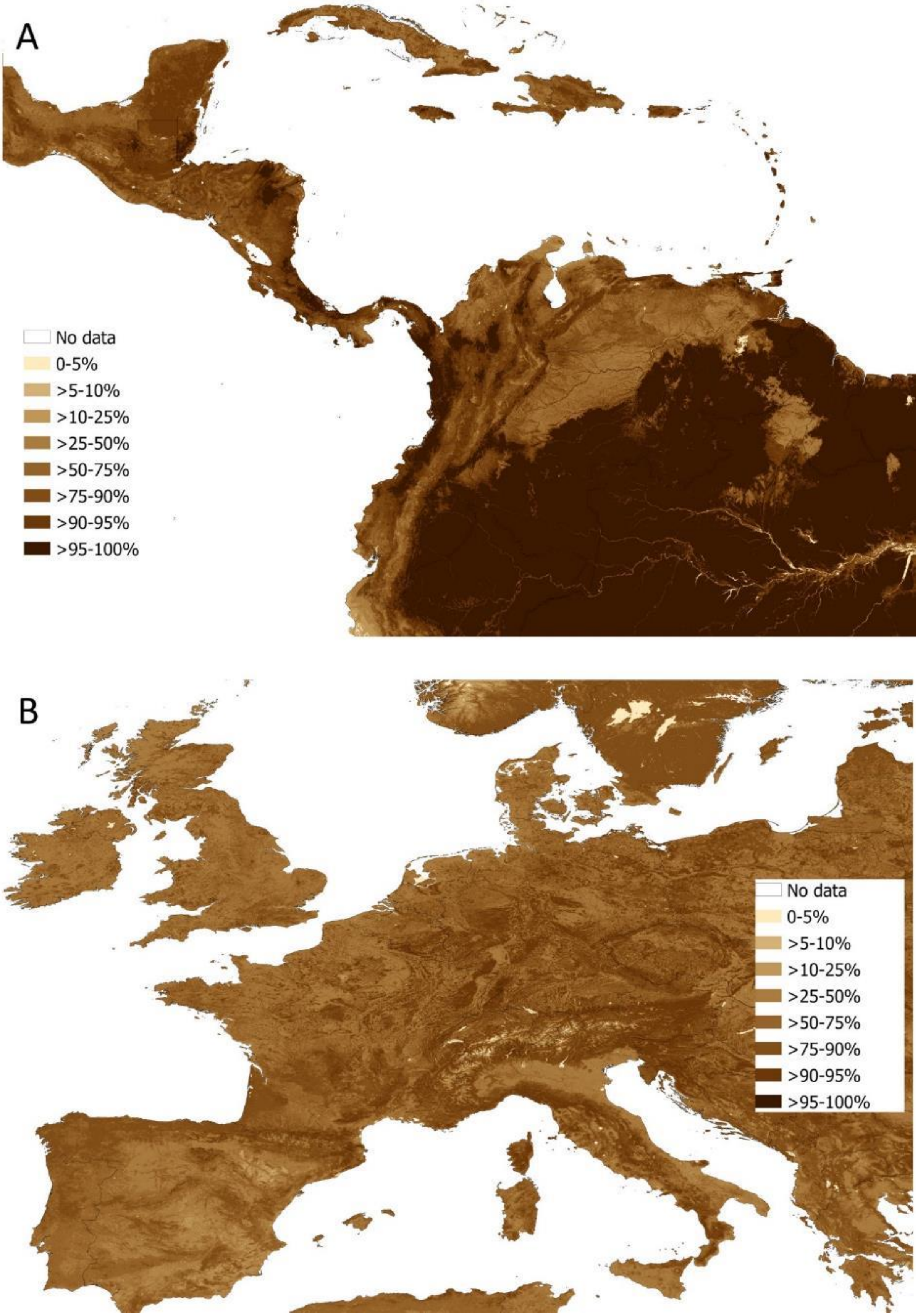

Figure S10. Example case studies for (A) Latin America (scale 1:16M) and (B) Europe (1:13M) for **above ground carbon**. True zero values (coloured) are distinguished from no-data (white).

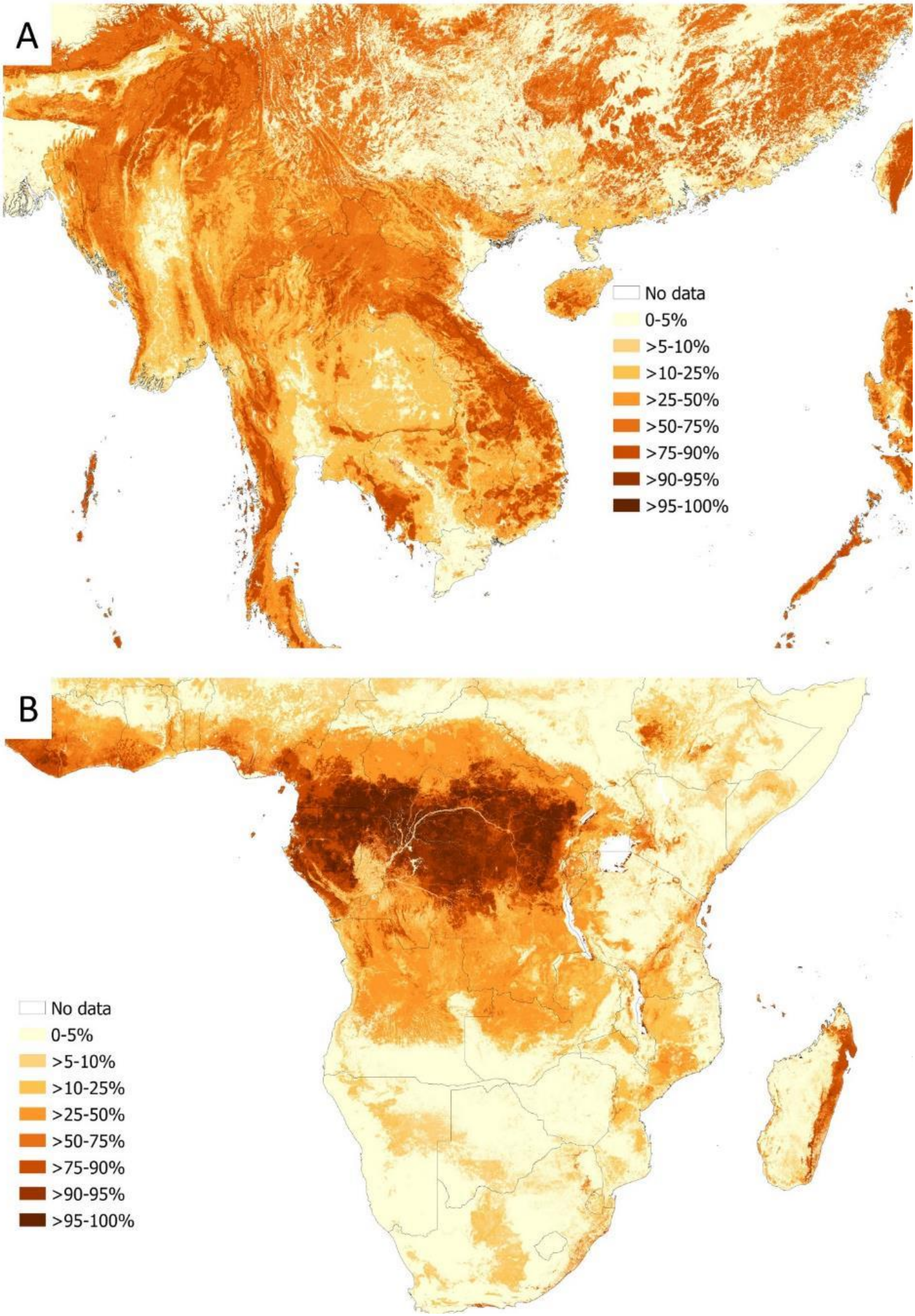

Figure S11. Example case studies for (A) South-Asia (scale 1:12M) and (B) Sub-Saharan Africa (1:25M) for **fuelwood production**. True zero values (coloured) are distinguished from no-data (white).

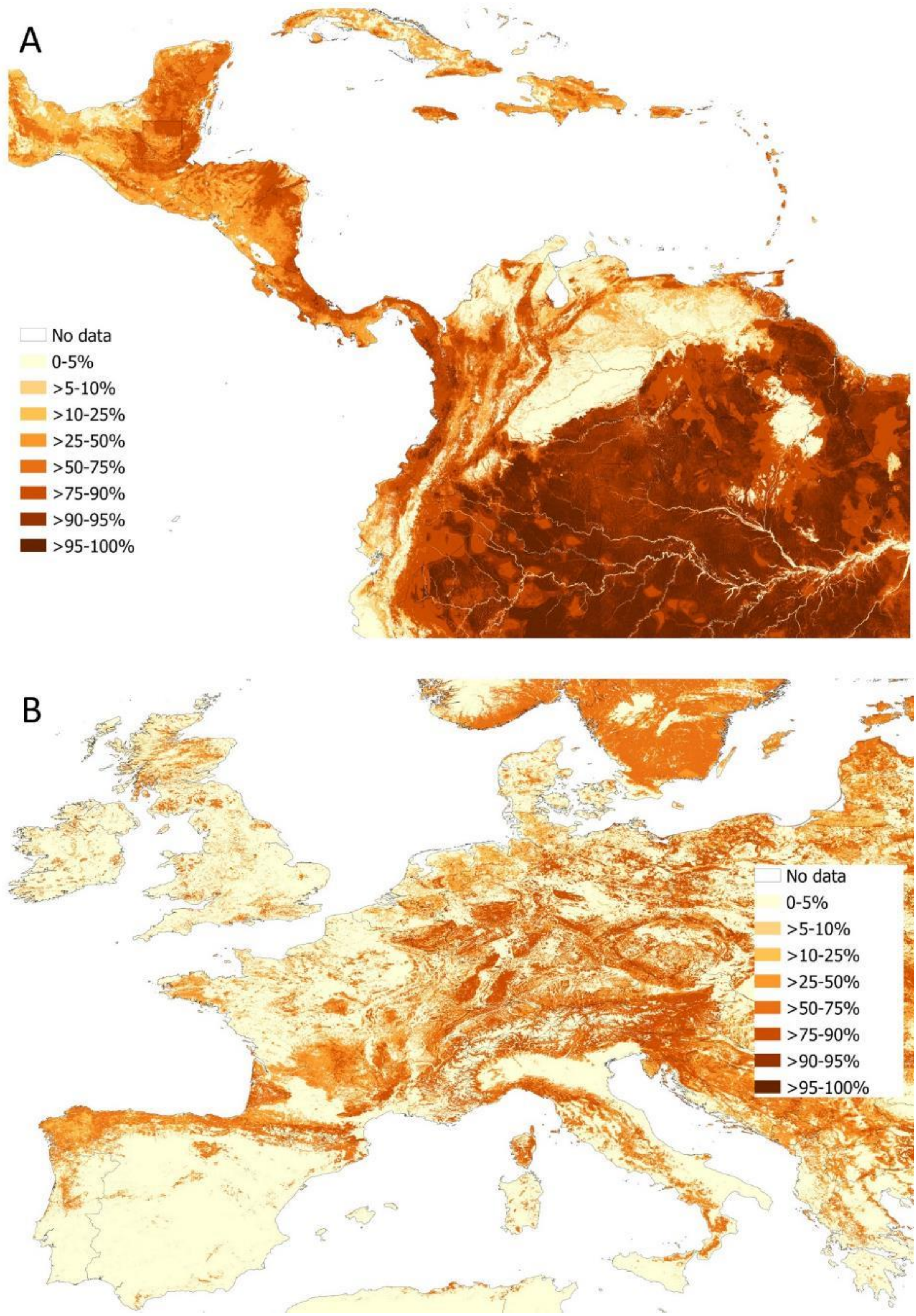

Figure S12. Example case studies for (A) Latin America (scale 1:16M) and (B) Europe (1:13M) for **fuelwood production**. True zero values (coloured) are distinguished from no-data (white).

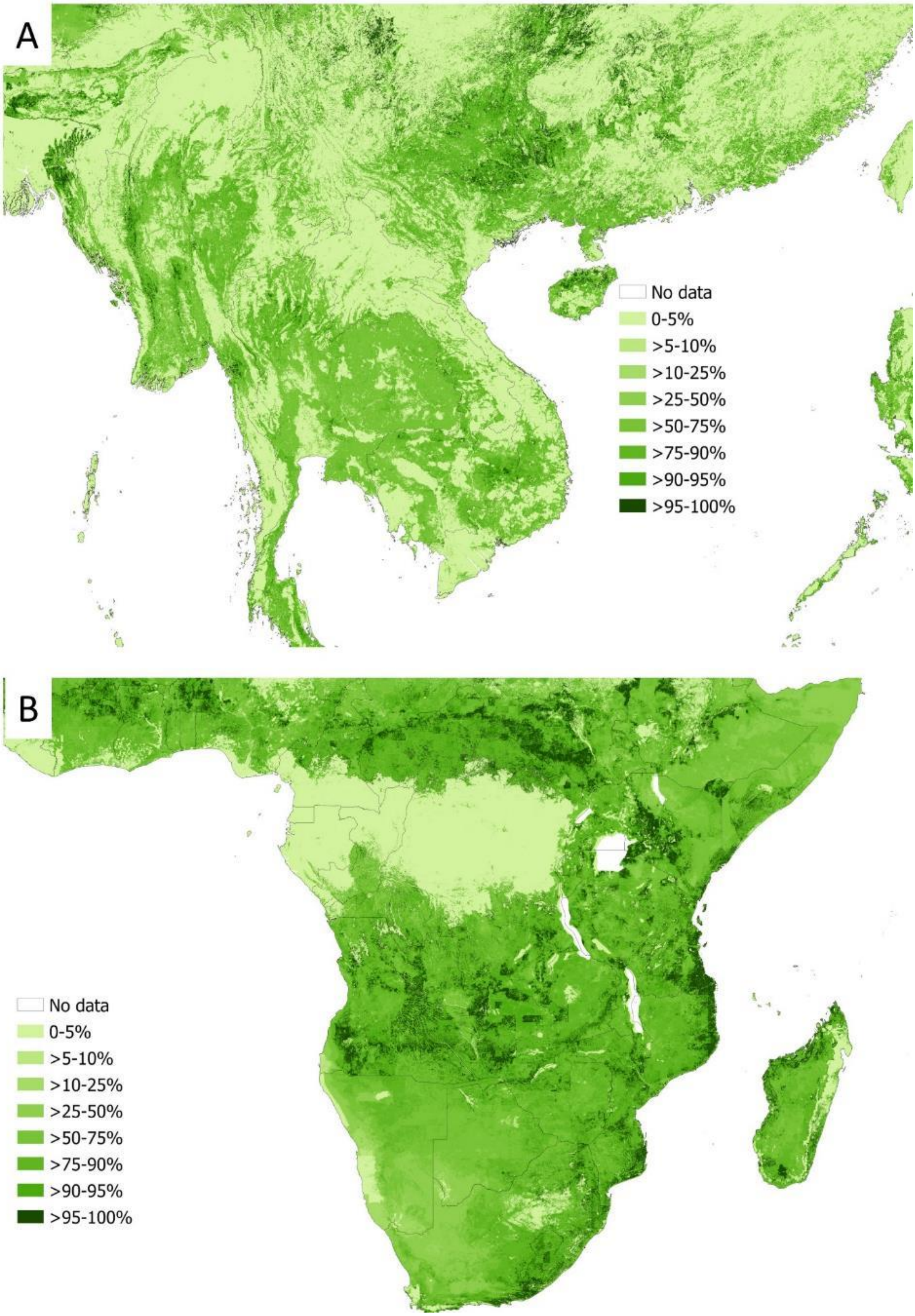

Figure S13. Example case studies for (A) South-Asia (scale 1:12M) and (B) Sub-Saharan Africa (1:25M) for **forage production**. True zero values (coloured) are distinguished from no-data (white).

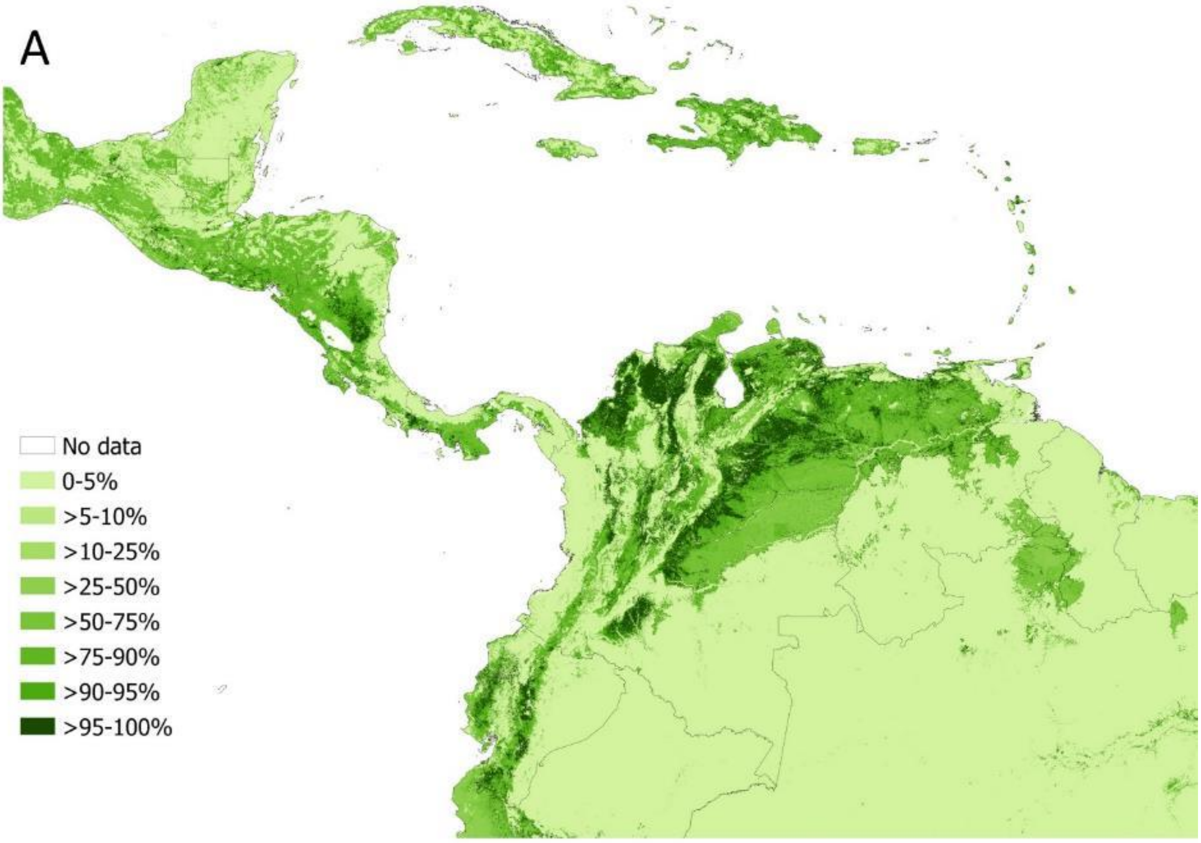

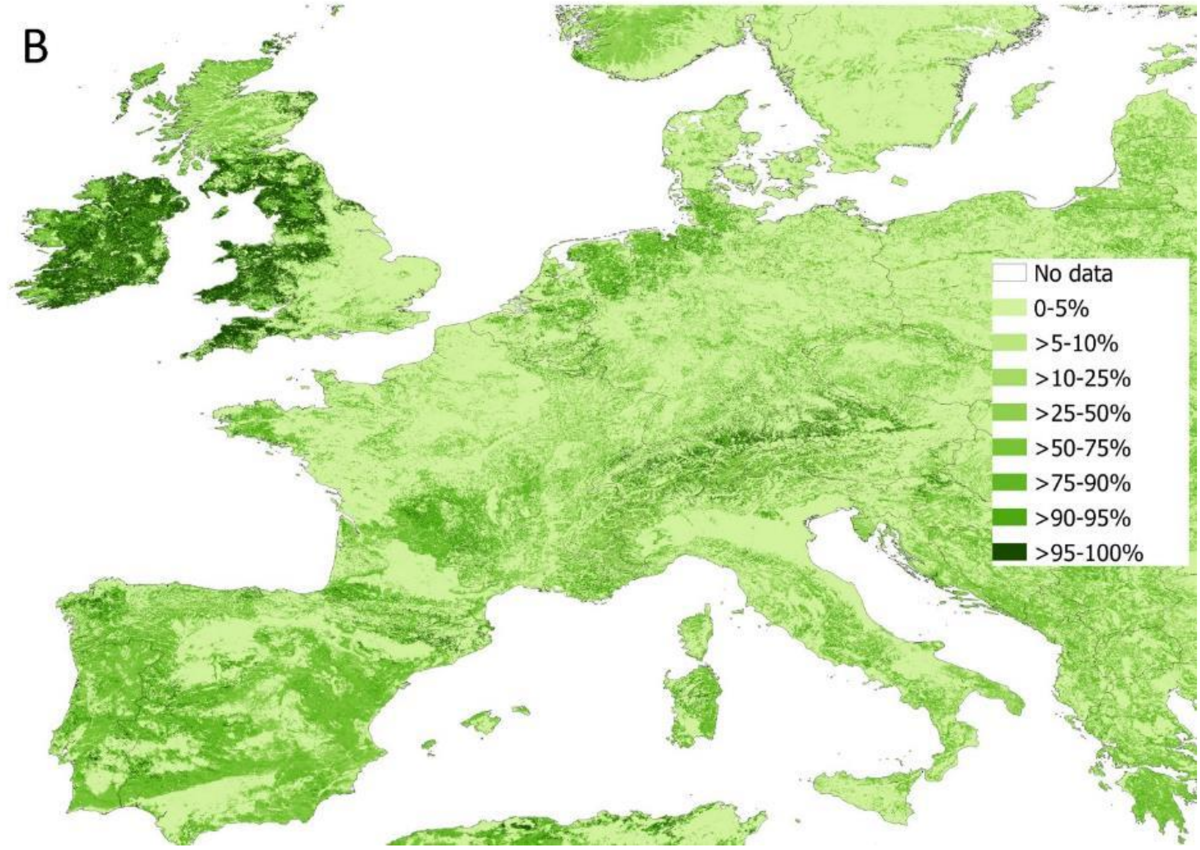

Figure S14. Example case studies for (A) Latin America (scale 1:16M) and (B) Europe (1:13M) for **forage production**. True zero values (coloured) are distinguished from no-data (white).

SI-7. Results with other ensemble approaches

In the main text we employ the median ensemble, for which the median value per cell or catchment polygon was used. Hooftman *et al.* (*20*) explored a wider variety of ensemble approaches, simulating alternative situations where reliable validation data are either present or lacking. Here we show the mean ensemble – i.e., taking the mean value per cell or catchment polygon. Generally the median ensemble shows a higher accuracy compared to the mean ensemble, which is more sensitive to outlier values (*13*, *20*). Furthermore, we show a selection of weighted approaches in which per cell the different models have a different product factor (see below). The weighted ensembles differ in how the weights are calculated. Two of the approaches shown are deterministic, which means that the result is an inherent property of the data set – i.e. the statistical outcome is identical given the same data set. Two approaches are iterative, which means that parameter space is step-wise systematically explored improving the maximum log-likelihood until `convergence is reached – i.e. no better solution is found. With iterative approaches, theoretically, different outcomes would be possible by redoing the calculation, however, the tools used are such that differences would be minimal. Both these deterministic and iterative approaches are based on searching for maximum consensus across input models. We also included an approach in which in which we penalised model outputs that are generated at coarser spatial resolutions (grain). See (*20*) for discussion of all approaches.

Weighted approaches often generate a good and reliable accuracy, with the disadvantage that they are more computationally intensive and are therefore not used in the main text. Weights in all cases were normalised to sum to 1 as $\left(\frac{\omega_i}{\sum_i^n \omega_i}\right)$, with weights ω_i for model *i* and *n* the total number of models per service. Due to bootstrapping among data-points, the resulting accuracy was different among runs, generating means and standard deviations.

Weighted approaches have the general form:

$$Ensemble_{(x)} = \sum_i^n \left(\frac{\omega_i}{\sum_i^n \omega_i} \times Y_i\right)_{(x)}$$

with positive weights ω_i for model *i* for cell/polygon *x*, weights ω_i are normalised to sum to 1, *Y* the modelled values for *i* per cell/polygon, and *n* the total number of models per service.

Approaches to determine weights that are used here:

1) PCA as the consensus axis is a deterministic consensus approach. Principal components were calculated using the Matlab *princomp*-tool, the weights per model *i* outputted to the equation above were the loadings to the first – main – PCA axis. So models with the better correlation to the consensus axis are assigned higher weights.
2) The correlation coefficient method is our second deterministic consensus approach. Here we calculated the full [model × model] correlation matrix using the *corrcoef*-tool. The weight per model was the mean correlation of that individual model with all other models, not including itself. Hence, the higher general correlation to the other models, the more weight a model has. This technique was developed to have a second deterministic approach using a consensus axis different than the PCA and can be seen as further way to minimise variance among models.
3) Regression to the median is our first iterative approach using log-likelihood regression (*31*). Using multivariate regression we assess weights such that the summed results maximises the explanation of an comparator. The resulting regression coefficients are used as weights and entered in the equation above. In this case the comparator is the median ensemble, asking which contribution of models would be most closely result to the median. The regression contains no constant, hence it can be represented as: [$E_{ii} \sim \omega_1 Y_1 + \omega_2 Y_2 .... + \omega_n Y_n$]. Multi-variate regression to the median was done using the *nlmefit*-tool, maximising log-likelihood with 200 iterations: repeating the regression 200 times), an output tolerance of $1.0000e^{-4}$ and naïve

priors (all $\omega_i = \frac{1}{n}$ at the start).

4) Leave-one-out cross-validation is our second iterative approach in which entire models are cross validated one-by-one. As for regression to the median this is done using a no constant multi-variation regression with the same *nlmefit*-tool as above, with the same settings and naïve priors. However, in this approach we loop through the model outputs. One-by-one, a regression is performed using a single model output as comparator and the remaining model outputs as explanatory variables. For model 1 such would be the regression representation [$Y_1 \sim \omega_2 Y_2 + \omega_3 Y_3 .... + \omega_n Y_n$]. The regression coefficients (ω_i) are stored as consensus weights. After looping through all models, the mean is taken of all regression coefficients per model as weights (excluding itself), *i.e.* this represents the weights that would generate the highest mean consensus with all models. These values are entered as weights in the equation above.
5) Models that are generated on smaller scales (*i.e.* with smaller gridcells) could be more accurate since the information per cell could better represent the local situation whereas larger gridcells could be more averaged across larger areas (*8*, *13*). Here, we penalised model outputs that are generated at coarser spatial resolutions (*14*). The grain size was taken from the original model outputs as downloaded or generated (see Table 2 main text), so before all post-processing. The weights taken were: $\omega_\mathrm{i} = \frac{1}{\log_{10}(\mathrm{grain_i})}$, for which the resulting weights were normalised afterwards summing to 1.

Below we present two accuracy metrics:

1) The inverse of deviance ($D^{\downarrow}$) – as used in the main text –, ascertaining the absolute difference of each modelled value from its validation value using the inverse of the deviance. This is relevant where modelled values are important, e.g., when testing where ES levels exceed a minimum threshold. We used the inverse of the deviance so that, like ρ, a higher value indicated greater accuracy. The calculation follows

$$D^{\downarrow} = 1 - \left(\frac{1}{n} \times \sum_x^n \left|X_{(x)} - Y_{(x)}\right|\right)$$

in which, n = the number of spatial data points, x a spatial data point, X(x) the normalised validation value for x, and Y(x) the normalised value for the model or ensemble tested.
2) Comparing the rank order of predicted and validation data using Spearman ρ using the Matlab *corr* tool. This is relevant where modelling is used to discover, for example, the most important locations for delivering an ES, or conversely, those areas whose development may have least impact on ES delivery.

To avoid directional confusion, when these metrics are used per point (Table 1 main text, Tables S7-9), we include an inverse of the per data point value for both deviance and ρ, i.e., the [1-value] applies to the data points; whereas in the full data-set value above it is a post-summation inverse. We note that the Spearman ρ as calculated under (2) incorporates an similar internal reverse as the inverse of the deviance, after summation of individual data point (high ranking differences providing a low ρ value).

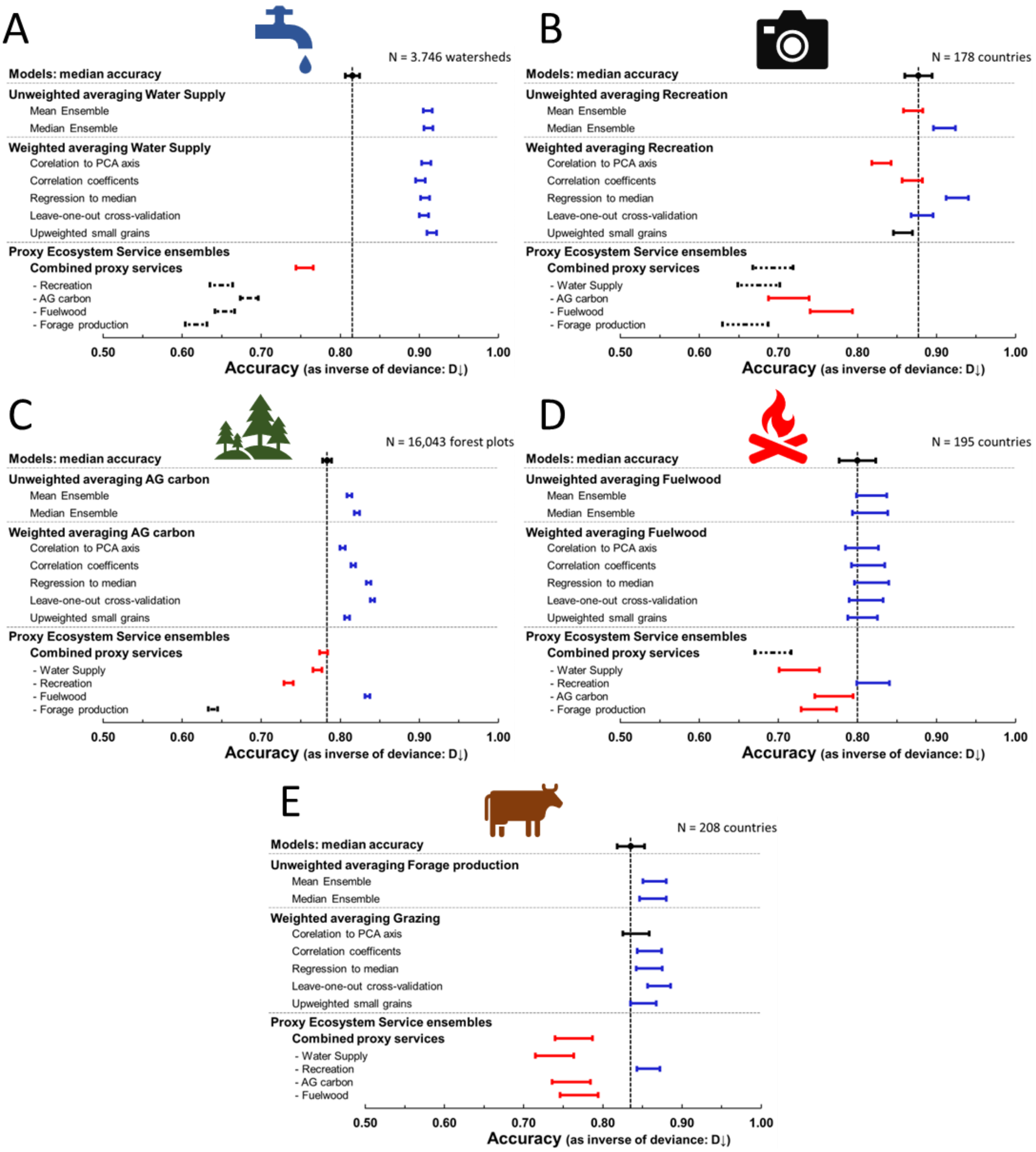

Figure S15. Ecosystem service ensembles show an increased inverse of the deviance as measure of accuracy ($D^{\downarrow}$) when compared to individual models with multiple ensemble methods from (*20*) for: a) water, b) recreation, c) AG carbon, d) fuelwood, e) forage production. Colours: significantly higher than mean accuracy of the models (blue), significantly lower than models (red), not significantly different from mean of the models (black) and $D^{\downarrow} < 0.7$ (dashed), a threshold for a 'good' explanation (*8*). Note meaning of black and dashed colouring slightly differ from the main text.

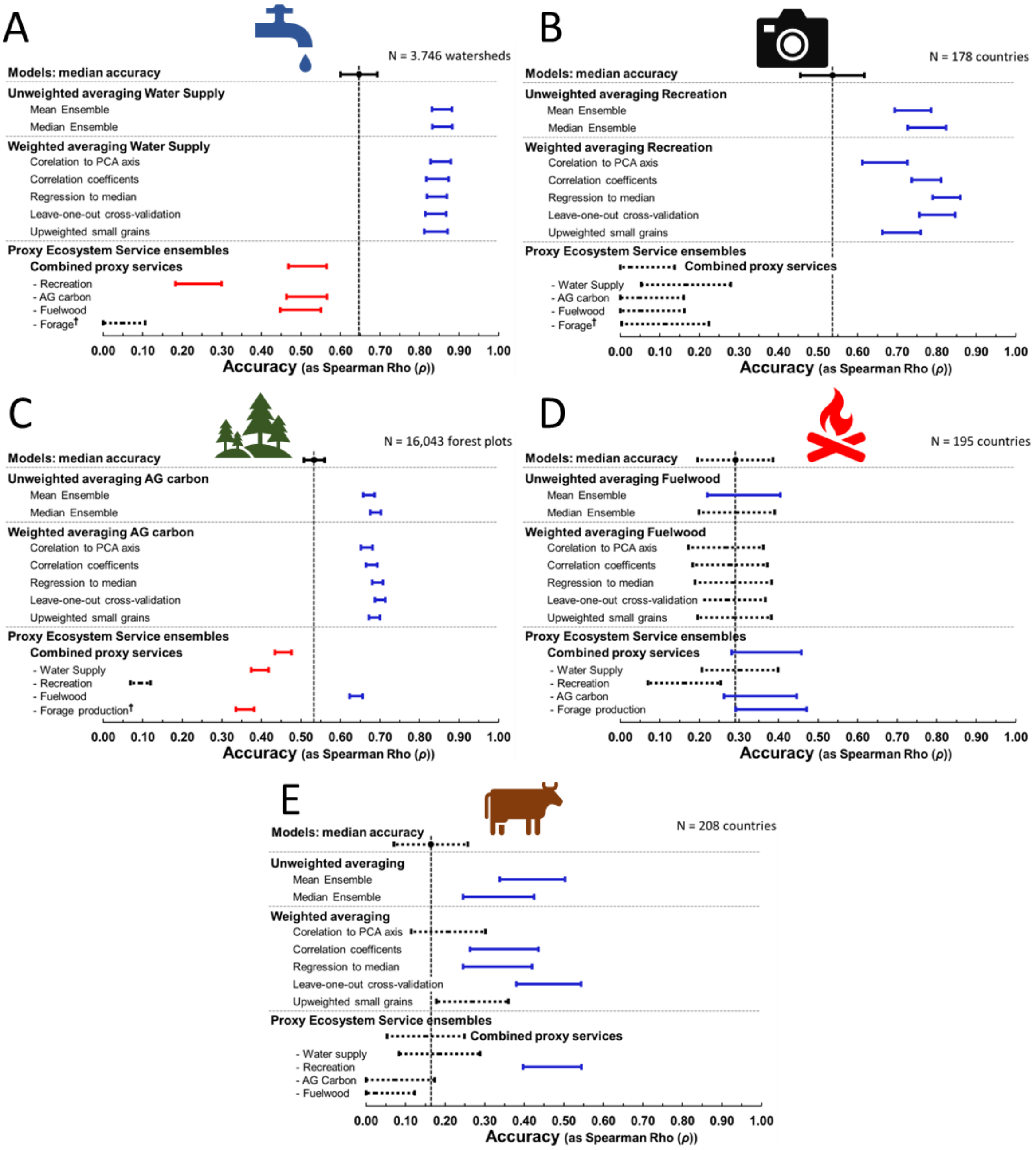

Figure S16. Ecosystem service ensembles show an increased Spearman ρ (ranking correlation) as measure of accuracy when compared to individual models with multiple ensemble methods from (*20*) for: a) water, b) recreation, c) AG carbon, d) fuelwood, e) forage production. Colours: significantly higher than mean accuracy of the models (blue), significantly lower than models (red), not significantly different from mean of the models (black) and no significant ranking correlation (dashed black). †opposite relationship (negative ρ).

Acknowledgments

This work took place under the following UKRI grants: NE/W005050/1, NE/T00391X/1, ES/R009279/1, ES/T007877/1, ES/V004077/1, ES/R006865/1, as well as UKCEH project 08695 and NSF GRFP Grant number DGE-2139899. JML was funded by a María Zambrano postdoctoral grant by the Spanish Ministry of Universities and the Next Generation European Union funds. SW, DAPH, RAN, RCH & JMB conceived the project. DAPH, JIB, TH, GK, ARL, ML, & JML provided ES model descriptions and outputs. DAPH conducted all analyses. SW, DAPH, RAN, RCH & JMB wrote the manuscript, with comments from all other authors. The authors declare that they have no competing interests. We also thank the anonymous reviewers for their insightful comments on the manuscript. All data needed to evaluate the conclusions in the paper are present in the paper and/or the Supplementary Materials.

REFERENCES AND NOTES

1. R. Chaplin-Kramer, R. P. Sharp, C. Weil, E. M. Bennett, U. Pascual, K. K. Arkema, K. A. Brauman, B. P. Bryant, A. D. Guerry, N. M. Haddad, M. Hamann, P. Hamel, J. A. Johnson, L. Mandle, H. M. Pereira, S. Polasky, M. Ruckelshaus, M. R. Shaw, J. M. Silver, A. L. Vogl, G. C. Daily, Global modeling of nature's contributions to people. *Science* **366**, 255–258 (2019).

2. R. Chaplin-Kramer, R. A. Neugarten, R. P. Sharp, P. M. Collins, S. Polasky, D. Hole, R. Schuster, M. Strimas-Mackey, M. Mulligan, C. Brandon, S. Diaz, E. Fluet-Chouinard, L. J. Gorenflo, J. A. Johnson, C. M. Kennedy, P. W. Keys, K. Longley-Wood, P. B. McIntyre, M. Noon, U. Pascual, C. Reidy Liermann, P. R. Roehrdanz, G. Schmidt-Traub, M. R. Shaw, M. Spalding, W. R. Turner, A. van Soesbergen, R. A. Watson, Mapping the planet's critical natural assets. *Nat. Ecol. Evol.* **7**, 51–61 (2023).

3. C. Brandon, K. Brandon, A. Fairbrass, R. Neugarten, Integrating natural capital into national accounts: Three decades of promise and challenge. *Rev. Environ. Econ. Policy.* **15**, 134–153 (2021).

4. R. Chaplin-Kramer, K. A. Brauman, J. Cavender-Bares, S. Díaz, G. T. Duarte, B. J. Enquist, L. A. Garibaldi, J. Geldmann, B. S. Halpern, T. W. Hertel, C. K. Khoury, J. M. Krieger, S. Lavorel, T. Mueller, R. A. Neugarten, J. Pinto-Ledezma, S. Polasky, A. Purvis, V. Reyes-García, P. R. Roehrdanz, L. J. Shannon, M. R. Shaw, B. B. N. Strassburg, J. M. Tylianakis, P. H. Verburg, P. Visconti, N. Zafra-Calvo, Conservation needs to integrate knowledge across scales. *Nat. Ecol. Evol.* **6**, 118–119 (2022).

5. C. Wyborn, M. C. Evans, Conservation needs to break free from global priority mapping. *Nat. Ecol. Evol.* **5**, 1322–1324 (2021).

6. IOS, ISO 5725-1:1994 Accuracy (trueness and precision) of measurement methods and results - Part 1: General principles and definitions (1994); available at https://www.iso.org/standard/11833.html.

7. W. E. Walker, P. Harremoës, J. Rotmans, J. P. van der Sluijs, M. B. A. van Asselt, P. Janssen, M. P. Krayer von Krauss, Defining uncertainty: A conceptual basis for uncertainty management in model-based decision support. *Integr. Assess.* **4**, 5–17 (2003).

8. S. Willcock, D. A. P. Hooftman, S. Balbi, R. Blanchard, T. P. Dawson, P. J. O'Farrell, T. Hickler, M. D. Hudson, M. Lindeskog, J. Martinez-Lopez, M. Mulligan, B. Reyers, C. Shackleton, N. Sitas, F. Villa, S. M. Watts, F. Eigenbrod, J. M. Bullock, A continental-scale validation of ecosystem service models. *Ecosystems* **22**, 1902–1917 (2019).

9. R. A. Neugarten, M. Honzak, P. Carret, K. Koenig, L. Andriamaro, C. A. Cano, H. S. Grantham, D. Hole, D. Juhn, M. McKinnon, A. Rasolohery, M. Steininger, T. M. Wright, W. R. Turner, Rapid assessment of ecosystem service co-benefits of biodiversity priority areas in Madagascar. *PLOS ONE* **11**, e0168575 (2016).

10. G. Schmidt-Traub, National climate and biodiversity strategies are hamstrung by a lack of maps. *Nat. Ecol. Evol.* **5**, 1325–1327 (2021).

11. L. A. Bruijnzeel, M. Mulligan, F. N. Scatena, Hydrometeorology of tropical montane cloud forests: Emerging patterns. *Hydrol. Process.* **25**, 465–498 (2011).

12. J. W. Redhead, L. May, T. H. Oliver, P. Hamel, R. Sharp, J. M. Bullock, National scale evaluation of the InVEST nutrient retention model in the United Kingdom. *Sci. Total Environ.* **610–611**, 666–677 (2018).

13. S. Willcock, D. A. P. Hooftman, R. Blanchard, T. P. Dawson, T. Hickler, M. Lindeskog, J. Martinez-Lopez, B. Reyers, S. M. Watts, F. Eigenbrod, J. M. Bullock, Ensembles of ecosystem service models can improve accuracy and indicate uncertainty. *Sci. Total Environ.* **747**, 141006 (2020).

14. S. Willcock, D. Hooftman, N. Sitas, P. O'Farrell, M. D. Hudson, B. Reyers, F. Eigenbrod, J. M. Bullock, Do ecosystem service maps and models meet stakeholders' needs? A preliminary survey across sub-Saharan Africa. *Ecosyst. Serv.* **18**, 110–117 (2016).

15. M. B. Araújo, M. New, Ensemble forecasting of species distributions. *Trends Ecol. Evol.* **22**, 42–47 (2007).

16. L. Olander, S. Polasky, J. S. Kagan, R. J. Johnston, L. Waigner, D. Saah, L. Maguire, J. Boyd, D. Yoskowitz, So you want your research to be relevant? Building the bridge between ecosystem services research and practice. *Ecosyst. Serv.* **26**, 170–182 (2017).

17. H. Suich, C. Howe, G. Mace, Ecosystem services and poverty alleviation: A review of the empirical links. *Ecosyst. Serv.* **12**, 137–147 (2015).

18. IPBES, E. S. Brondizio, J. Settele, S. Díaz, H. T. Ngo, Eds. (Bonn, Germany, 2019), pp. 1–1148; https://ipbes.net/global-assessment.

19. IPBES, in *Secretariat of the Intergovernmental Science-Policy Platform on Biodiversity and Ecosystem Services*, S. Ferrier, K. N. Ninan, P. Leadley, R. Alkemade, L. A. Acosta, H. R. Akçakaya, L. Brotons, W. W. L. Cheung, V.Christensen, K. A. Harhash, J. Kabubo-Mariara, C. Lundquist, M. Obersteiner, H. Pereira, G. Peterson, R. Pichs-Madruga, N. Ravindranath, C. Rondinini, B. A. Wintle, Eds. (Secretariat of the Intergovernmental Platform for Biodiversity and Ecosystem Services, Bonn, Germany, 2016), p. 370; https://www.ipbes.net/sites/default/files/downloads/pdf/2016.methodological_assessment_report_scenarios_models.pdf.

20. D. A. P. Hooftman, J. M. Bullock, L. Jones, F. Eigenbrod, J. I. Barredo, M. Forrest, G. Kindermann, A. Thomas, S. Willcock, Reducing uncertainty in ecosystem service modelling through weighted ensembles. *Ecosyst. Serv.* **53**, 101398 (2022).

21. K. He, L. Yu, K. K. Lai, Crude oil price analysis and forecasting using wavelet decomposed ensemble model. *Energy* **46**, 564–574 (2012).

22. B. P. Bryant, M. E. Borsuk, P. Hamel, K. L. L. Oleson, C. J. E. Schulp, S. Willcock, Transparent and feasible uncertainty assessment adds value to applied ecosystem services modeling. *Ecosyst. Serv.* **33**, 103–109 (2018).

23. F. Villa, K. J. Bagstad, B. Voigt, G. W. Johnson, R. Portela, M. Honzák, D. Batker, A methodology for adaptable and robust ecosystem services assessment. *PLOS ONE* **9**, e91001 (2014).

24. R. Sharp, J. Douglass, S. Wolny, K. Arkema, J. Bernhardt, W. Bierbower, N. Chaumont, D. Denu, D. Fisher, K. Glowinski, R. Griffin, G. Guannel, A. Guerry, J. Johnson, P. Hamel, C. Kennedy, C. K. Kim, M. Lacayo, E. Lonsdorf, L. Mandle, L. Rogers, J. Silver, J. Toft, G. Verutes, A. L. Vogl, S. Wood, K. Wyatt, *InVEST 3.11.0.post88+ug.gbbddbb6 User's Guide* (The Natural Capital Project; Stanford

University: Stanford, CA, USA; University of Minnesota: Minneapolis, MN, USA; The Nature Conservancy: Arlington, VA, USA; World Wildlife Fund: Washington, DC, USA, 2020).

25. M. Mulligan, in *Impact of Climate Change on Water Resources in Agriculture*, C. Zolin, R. de A. R. Rodrigues, Eds. (CRC Press, 2015), pp. 184–204.

26. M. Potschin-Young, R. Haines-Young, C. Görg, U. Heink, K. Jax, C. Schleyer, Understanding the role of conceptual frameworks: Reading the ecosystem service cascade. *Ecosyst. Serv.* **29**, 428–440 (2018).

27. UK National Ecosystem Assessment, *The UK National Ecosystem Assessment: Synthesis of the Key Findings* (2011).

28. J. W. Redhead, C. Stratford, K. Sharps, L. Jones, G. Ziv, D. Clarke, T. H. Oliver, J. M. Bullock, Empirical validation of the InVEST water yield ecosystem service model at a national scale. *Sci. Total Environ.* **569-570**, 1418–1426 (2016).

29. R. Spake, R. Lasseur, E. Crouzat, J. M. Bullock, S. Lavorel, K. E. Parks, M. Schaafsma, E. M. Bennett, J. Maes, M. Mulligan, M. Mouchet, G. D. Peterson, C. J. E. Schulp, W. Thuiller, M. G. Turner, P. H. Verburg, F. Eigenbrod, Unpacking ecosystem service bundles: Towards predictive mapping of synergies and trade-offs between ecosystem services. *Glob. Environ. Chang.* **47**, 37–50 (2017).

30. T. Hao, J. Elith, J. J. Lahoz-Monfort, G. Guillera-Arroita, Testing whether ensemble modelling is advantageous for maximising predictive performance of species distribution models. *Ecography (Cop.)* **43**, 549–558 (2020).

31. C. F. Dormann, J. M. Calabrese, G. Guillera-Arroita, E. Matechou, V. Bahn, K. Bartoń, C. M. Beale, S. Ciuti, J. Elith, K. Gerstner, J. Guelat, P. Keil, J. J. Lahoz-Monfort, L. J. Pollock, B. Reineking, D. R. Roberts, B. Schröder, W. Thuiller, D. I. Warton, B. A. Wintle, S. N. Wood, R. O. Wüest, F. Hartig, Model averaging in ecology: A review of Bayesian, information-theoretic, and tactical approaches for predictive inference. *Ecol. Monogr.* **88**, 485–504 (2018).

32. H. Ding, J. M. Bullock, *A Guide to Selecting Ecosystem Service models for Decision-Making: Lessons from Sub-Saharan Africa* (2018); https://www.wri.org/research/guide-selecting-ecosystem-service-models-decision-making-lessons-sub-saharan-africa.

33. C. P. Wong, B. Jiang, A. P. Kinzig, K. N. Lee, Z. Ouyang, Linking ecosystem characteristics to final ecosystem services for public policy. *Ecol. Lett.* **18**, 108–118 (2015).

34. S. Willcock, J. Martínez-López, D. A. P. Hooftman, K. J. Bagstad, S. Balbi, A. Marzo, C. Prato, S. Sciandrello, G. Signorello, B. Voigt, F. Villa, J. M. Bullock, I. N. Athanasiadis, Machine learning for ecosystem services. *Ecosyst. Serv.* **33**, 165–174 (2018).

35. M. Mulligan, WaterWorld: A self-parameterising, physically based model for application in data-poor but problem-rich environments globally. *Hydrol. Res.* **44**, 748–769 (2013).

36. J. I. Barredo, J. San Miguel, G. Caudullo, L. Busetto, A European map of living forest biomass and carbon stock: Executive report, EUR 25730 EN (Luxembourg, 2012).

37. European Commission, Joint Research Centre, Forest Biomass Map of Europe (2020); available at http://data.europa.eu/89h/d1fdf7aa-df33-49af-b7d5-40d226ec0da3.

38. V. Avitabile, M. Herold, G. B. M. Heuvelink, S. L. Lewis, O. L. Phillips, G. P. Asner, J. Armston, P. S. Ashton, L. Banin, N. Bayol, N. J. Berry, P. Boeckx, B. H. J. de Jong, B. DeVries, C. A. J. Girardin, E. Kearsley, J. A. Lindsell, G. Lopez-Gonzalez, R. Lucas, Y. Malhi, A. Morel, E. T. A. Mitchard, L. Nagy, L. Qie, M. J. Quinones, C. M. Ryan, S. J. W. Ferry, T. Sunderland, G. V. Laurin, R. C. Gatti, R. Valentini, H. Verbeeck, A. Wijaya, S. Willcock, An integrated pan-tropical biomass map using multiple reference datasets. *Glob. Chang. Biol.* **22**, 1406–1420 (2016).

39. S. Quegan, T. Le Toan, J. Chave, J. Dall, J.-F. Exbrayat, D. H. T. Minh, M. Lomas, M. M. D'Alessandro, P. Paillou, K. Papathanassiou, F. Rocca, S. Saatchi, K. Scipal, H. Shugart, T. L. Smallman, M. J. Soja, S. Tebaldini, L. Ulander, L. Villard, M. Williams, The European space agency BIOMASS mission: Measuring forest above-ground biomass from space. *Remote Sens. Environ.* **227**, 44–60 (2019).

40. N. L. Harris, D. A. Gibbs, A. Baccini, R. A. Birdsey, S. de Bruin, M. Farina, L. Fatoyinbo, M. C. Hansen, M. Herold, R. A. Houghton, P. V. Potapov, D. R. Suarez, R. M. Roman-Cuesta, S. S. Saatchi, C. M. Slay, S. A. Turubanova, A. Tyukavina, Global maps of twenty-first century forest carbon fluxes. *Nat. Clim. Chang.* **11**, 234–240 (2021).

41. A. Jarvis, H. I. Reuter, A. Nelson, E. Guevara, Hole-filled seamless SRTM data V4 (2008); available at http://srtm.csi.cgiar.org.

42. M. Schläpfer, L. Dong, K. O'Keeffe, P. Santi, M. Szell, H. Salat, S. Anklesaria, M. Vazifeh, C. Ratti, G. B. West, The universal visitation law of human mobility. *Nature* **593**, 522–527 (2021).

43. M. Gilbert, G. Nicolas, G. Cinardi, T. P. Van Boeckel, S. Vanwambeke, W. G. R. Wint, T. P. Robinson, Gridded Livestock of the World in 2010 (5 minutes of arc) V3 (2018); available at https://dataverse.harvard.edu/dataverse/glw_3.

44. P. D. Broxton, X. Zeng, D. Sulla-Menashe, P. A. Troch, A global land cover climatology using MODIS data. *J. Appl. Meteorol. Climatol.* **53**, 1593–1605 (2014).

45. R. Costanza, R. de Groot, P. Sutton, S. van der Ploeg, S. J. Anderson, I. Kubiszewski, S. Farber, R. K. Turner, Changes in the global value of ecosystem services. *Glob. Environ. Chang.* **26**, 152–158 (2014).

46. B. Lehner, K. L. Verdin, A. Jarvis, New global hydrography derived from spaceborne elevation data. *Eos (Washington. DC).* **89**, 93–94 (2008).

47. A. Grainger, Difficulties in tracking the long-term global trend in tropical forest area. *Proc. Natl. Acad. Sci. U.S.A.* **105**, 818–823 (2008).

48. S. Eker, E. Rovenskaya, M. Obersteiner, S. Langan, Practice and perspectives in the validation of resource management models. *Nat. Commun.* **9**, 5359 (2018).

49. Y. Huang, J. C. Hsu, Hochberg's step-up method: Cutting corners off Holm's step-down method. *Biometrika* **94**, 965–975 (2007).

50. E. A. Stanton, The Human Development Index: A History. Working Paper Series Number 127 (Amherst, USA, 2007); available at www.peri.umass.edu.

51. C. F. Dormann, J. M. McPherson, M. B. Araújo, R. Bivand, J. Bolliger, G. Carl, R. G. Davies, A. Hirzel, W. Jetz, W. Daniel Kissling, I. Kühn, R. Ohlemüller, P. R. Peres-Neto, B. Reineking, B.

Schröder, F. M. Schurr, R. Wilson, Methods to account for spatial autocorrelation in the analysis of species distributional data: A review. *Ecography* **30**, 609–628 (2007).

52. A. Ruesch, H. K. Gibbs, New IPCC Tier1 Global Biomass Carbon Map For the Year 2000. *Available online from Carbon Dioxide Inf. Anal. Cent. [http//cdiac.ornl.gov/ - accessed 15/01/12], Oak Ridge Natl. Lab. Oak Ridge, Tennessee.* (2008).

53. B. Smith, D. Wårlind, A. Arneth, T. Hickler, P. Leadley, J. Siltberg, S. Zaehle, Implications of incorporating N cycling and N limitations on primary production in an individual-based dynamic vegetation model. *Biogeosciences* **11**, 2027–2054 (2014).

54. R. J. Scholes, The South African 1: 250 000 Maps of Areas of Homogeneous Grazing Potential (1998).

55. F. Gassert, M. Landis, M. Luck, P. Reig, T. Shiao, Aqueduct Global Maps 2.1. (World Resources Institute, 2014).

56. M. L. Noon, A. Goldstein, J. C. Ledezma, P. R. Roehrdanz, S. C. Cook-Patton, S. A. Spawn-Lee, T. M. Wright, M. Gonzalez-Roglich, D. G. Hole, J. Rockström, W. R. Turner, Mapping the irrecoverable carbon in Earth's ecosystems. *Nat. Sustain.* **5**, 37–46 (2022).

57. G. E. Kindermann, I. McCallum, S. Fritz, M. Obersteiner, A global forest growing stock, biomass and carbon map based on FAO statistics. *Silva Fenn.* **42**, 387–396 (2008).

58. S. A. Spawn, C. C. Sullivan, T. J. Lark, H. K. Gibbs, Harmonized global maps of above and belowground biomass carbon density in the year 2010. *Sci. Data* **7**, 112 (2020).

59. J. H. Goldstein, G. Caldarone, T. K. Duarte, D. Ennaanay, N. Hannahs, G. Mendoza, S. Polasky, S. Wolny, G. C. Daily, Integrating ecosystem-service tradeoffs into land-use decisions. *Proc. Natl. Acad. Sci. U.S.A.* **109**, 7565–70 (2012).

60. C. Byczek, P. Y. Longaretti, J. Renaud, S. Lavorel, Benefits of crowd-sourced GPS information for modelling the recreation ecosystem service. *PLOS ONE* **13**, e0202645 (2018).

61. K. E. Saxton, W. J. Rawls, Soil water characteristic estimates by texture and organic matter for hydrologic solutions. *Soil Sci. Soc. Am. J.* **70**, 1569–1578 (2006).

62. C. DiMiceli, M. Carroll, R. Sohlberg, D.-H. Kim, M. Kelly, J. Townshend, MOD44B MODIS/Terra Vegetation Continuous Fields Yearly L3 Global 250m SIN Grid V006 (NASA EOSDIS Land Processes DAAC, 2015).

63. R. de Groot, L. Brander, S. van der Ploeg, R. Costanza, F. Bernard, L. Braat, M. Christie, N. Crossman, A. Ghermandi, L. Hein, S. Hussain, P. Kumar, A. McVittie, R. Portela, L. C. Rodriguez, P. ten Brink, P. van Beukering, Global estimates of the value of ecosystems and their services in monetary units. *Ecosyst. Serv.* **1**, 50–61 (2012).

64. FAO, Guidelines for the Measurement of Productivity and Efficiency in Agriculture (2018); available at https://www.fao.org/3/ca6395en/ca6395en.pdf.

65. S. L. Lewis, G. Lopez-Gonzalez, B. Sonké, K. Affum-Baffoe, T. R. Baker, L. O. Ojo, O. L. Phillips, J. M. Reitsma, L. White, J. A. Comiskey, M.-N. Djuikouo, C. E. N. Ewango, T. R. Feldpausch, A. C. Hamilton, M. Gloor, T. Hart, A. Hladik, J. Lloyd, J. C. Lovett, J.-R. Makana, Y. Malhi, F. M. Mbago, H. J. Ndangalasi, J. Peacock, K. S.-H. Peh, D. Sheil, T. Sunderland, M. D. Swaine, J. Taplin, D. Taylor, S. C. Thomas, R. Votere, H. Wöll, Increasing carbon storage in intact African tropical forests. *Nature* **457**, 1003–1006 (2009).
